# Synthesis and Properties of Silver Nanoparticles Functionalized with β-Cyclodextrin and Their Loading with Lupinine and Its Acetyl Derivatives

**DOI:** 10.3390/molecules30163354

**Published:** 2025-08-12

**Authors:** Serik D. Fazylov, Zhangeldy S. Nurmaganbetov, Oralgazy A. Nurkenov, Akmaral Z. Sarsenbekova, Olzhas T. Seilkhanov, Roza B. Seidakhmetova, Anel Z. Mendibayeva, Ryszhan Y. Bakirova, Zainulla M. Muldakhmetov

**Affiliations:** 1 Laboratory of Synthesis of Biologically Active Substances, Institute of Organic Synthesis and Coal Chemistry of the Republic of Kazakhstan, Karaganda 100008, Kazakhstan; iosu8990@mail.ru (S.D.F.); nurkenov_oral@mail.ru (O.A.N.); anenyawa@mail.ru (A.Z.M.); iosu.rk@mail.ru (Z.M.M.); 2 Department of Physical and Analytical Chemistry, Karaganda University of the Name of E.A. Buketov, Karaganda 100074, Kazakhstan; chem_akmaral@mail.ru; 3 Laboratory of Engineering Profile NMR-Spectroscopy, Sh. Ualikhanov Kokshetau University, Kokshetau 120000, Kazakhstan; seilkhanov@mail.ru; 4 Department of Clinical Pharmacology and Evidence-Based Medicine, Karaganda Medical University, Karaganda 100012, Kazakhstan; rozabat@mail.ru; 5 Department of Internal Diseases, Karaganda Medical University, Karaganda 100012, Kazakhstan; bakir15@mail.ru

**Keywords:** lupinine, lupinyl acetate, silver nanoparticles, bactericidal, cytotoxic, analgetic activities, nanocomposite, 2-hydroxypropyl-β-cyclodextrin

## Abstract

This study presents the results of a study of the synthesis and properties of 2-hydroxy-β-cyclodextrin functionalized by silver nanoparticles and its loading with a bioactive component. As a reducing agent and stabilizer, 2-Hydroxy-β-cyclodextrin (2gβCD) was used in the production of silver nanoparticles. The use of 2gβCD-AgNPs in loading molecules of the plant alkaloid lupinine (Lup) and its acetyl derivative (Lac) with bactericidal properties were studied. The formation of Lup-2gβCD-AgNPs and Lac-2gβCD-AgNPs was confirmed by UV spectroscopy and X-ray diffraction spectroscopy (XRD). Transmission electron microscopy (TEM) showed that the synthesized AgNPs had a spherical shape. ^1^H-, ^13^C-NMR nuclear magnetic resonance spectroscopy and Fourier transform infrared spectroscopy (FT-IR) confirmed the reduction and encapsulation of AgNPs by 2gβCD. Thermographic data show that the obtained Lup and its derivative inclusion complexes reduced energy barriers. This makes them promising components for thermosensitive functional materials. Encapsulated complexes of Lup and its acetate inclusion with silver nanoparticles demonstrated significantly (*p* < 0.05) higher antibacterial, cytotoxic, and moderately pronounced analgesic activity.

## 1. Introduction

Lupinine (Lup) is a simple representative of a large group of quinolizidine alkaloids found in plants of the genera Lupinus and Anabasis [1,2]. By their pharmacological action, Lup and its derivatives have bactericidal, sedative effects [3,4] and anti-inflammatory, antiviral properties [2,4]. They are of interest as promising pharmacophores [5]. The possibilities of modifying the structure of the Lup molecule enable the synthesis of new compounds and the study of their biological properties [6,7,8]. Lup ethers are the most studied of its known derivatives. For instance, a number of vinyl ethers of Lup showed antibacterial and local anesthetic effects [9,10,11,12] and anticholinesterase activity [11,12,13,14]. For this reason, it is of interest to obtain a composition of Lup and its acetate derivative with silver nanoparticles to study the synergistic effect in their manifestation of antibacterial and other properties.

Recent advances in nanotechnology have led to the development of nanomaterials with unique physicochemical and biological properties, enabling a wide range of biomedical applications [15,16]. Nanocomposite materials containing silver nanoparticles (AgNPs) have unique properties. They are used in medicine as effective antibacterial and antiviral agents [17,18]. Silver-containing materials exhibit pronounced antibacterial activity, making them valuable in both medical and environmental contexts. In medicine, they are used to reduce infection rates during burn treatment [19,20], to prevent bacterial colonization on catheter surfaces [21], and to eliminate microorganisms on textile materials [22]. Furthermore, their disinfectant properties are applied in water treatment processes [23]. In addition, it has also been reported in the literature that AgNPs exhibit strong cytoprotective activity against infections caused by the human immunodeficiency virus (HIV) [24]. The biological properties of (AgNPs), along with their size, distribution, and stability, depend largely on the nature of the reducing agents and the stabilizing polymer matrix. These characteristics are also influenced by the specific conditions under which the nanoparticles are formed within the composite. Various natural (oligosaccharides, cellulose, gelatin, etc.) and synthetic (polyvinylpyrrolidone, polyvinyl alcohol) polymers can be used as a stabilizing polymer matrix for AgNPs [25,26,27]. Environmentally and biologically hazardous reducing agents such as sodium borohydride and formaldehyde were also widely used as reducing agents earlier. In recent years, α-, β- and γ-CDs (α-, β- and γ-CD), cyclic oligosaccharides derived from natural starch, have gained wide application in both pharmacology and food technology. Their primary functions include enhancing the solubility and chemical stability of various bioactive compounds, as well as extending their shelf life [28]. Thus, all of the above stimulated the preparation and comprehensive study of water-soluble nanocomposites consisting of encapsulated Lup and Lupinyl acetate (Lac) in a 2gβCD matrix with nanoscale silver particles.

## 2. Results and Discussion

### 2.1. Structural Characteristics of Lup(Lac)-2gβCD-AgNPs Nanocomposites

**Figure 1:**
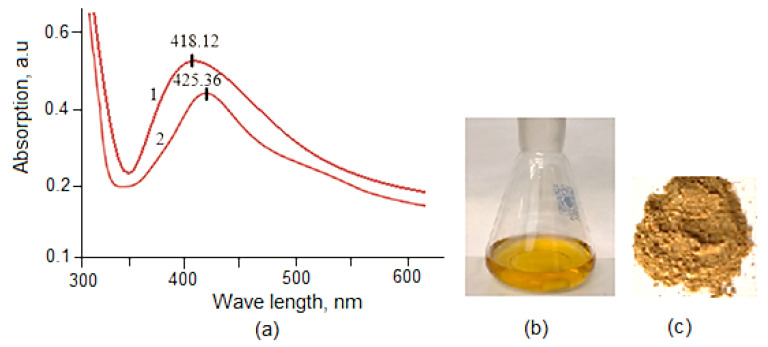
(**a**)-UV-vis spectrum of Lup(Lac)-2gβCD-AgNPs; (**b**)-the solution of Lup-2gβCD-AgNPs; (**c**)-dry powder Lup-2gβCD-AgNPs.

The formation of Lup (Lac)-2gβCD-AgNPs in the studied media was confirmed by UV-visible spectroscopy. This was evidenced by the appearance of characteristic surface plasmon resonance (SPR) bands, as shown in Figure 1a–c. These observations show that as the reaction time increases, the particle size and aggregation of silver nanocrystals gradually increase. All further measurements were carried out at room temperature (20 ± 0.05 °C). The absorption spectra of 2gβCD-AgNPs in the UV range at 418.12 nm indicate that the obtained nanoparticles have an absorption peak characteristic of spherical nanoparticles (Figure 1a) [29]. Nanocomposites were isolated from the resulting colored sol (Figure 1c). The resulting nanocomposites were yellow-brown powders, soluble in water. The analysis of the elemental data showed that the silver content in the nanocomposites is 3.5–4.0%. Obtaining a supramolecular complex of Lup and its acetate (Figure 1) will allow the development of their new water-soluble bioactive form.

**Figure 2:**
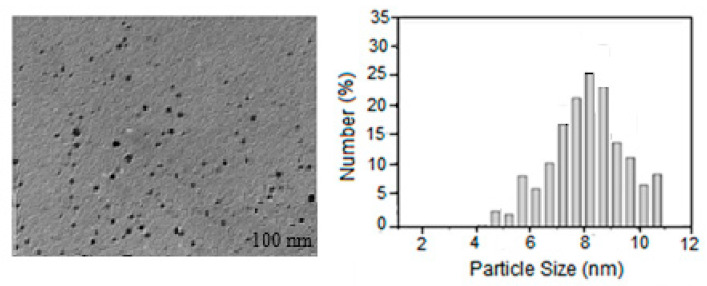
TEM images and size distribution of the AgNPs nanoparticles. Average particle size is 8.5 ± 1.14 nm, 60 min, pH = 9.25.

Previous studies showed that spherical AgNPs contribute to absorption bands of about 400 nm in the UV-visible spectrum [16,17,18]. This study revealed the surface plasmon resonance (SPR) bands of AgNPs at 418.12 nm for Lup and 425.36 nm for Lac (Figure 1a,b). These results strongly suggest that the AgNPs possess a spherical shape. This conclusion was further confirmed by transmission electron microscopy (TEM) analysis conducted in this study. TEM studies were performed to monitor the morphology, size, and dispersion of the obtained Lup-2gβCD-AgNPs. Analysis of the TEM image (Figure 2) showed that Lup-2gβCD-AgNPs have mainly a subspherical shape. The histogram demonstrates a narrow particle size distribution in the solution at pH 9.25. Most particles were within the 8–15 nm range, with an average size of 8.5 ± 1.17 nm.

**Figure 3:**
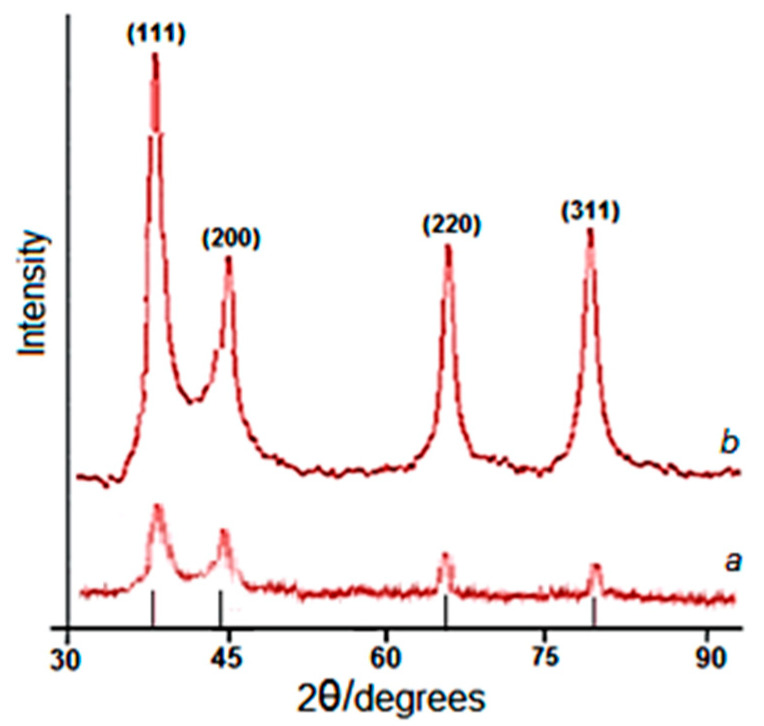
XRD pattern of Lup-2gβCD-AgNPs (a—60 min after the start of the reaction; b—after 120 min from the start of the Ag^+^ reduction reaction).

The histograms clearly show an increase in particle size with increasing time. The observed pattern indicates a slow growth of Ag nanoparticles in the matrix of the 2gβCD solution. Analysis of the temporal evolution of particle size distribution suggests clear size selectivity in the reaction. The preferred particle sizes were predominantly in the range of 6-11 nm. The synthesized nanoparticles were also characterized by X-ray diffraction (Figure 3). The X-ray diffraction (XRD) peaks confirmed that the AgNPs possessed a face-centered cubic crystal structure. No peaks corresponding to impurity crystalline phases were observed. Figure 3 shows X-ray images of AgNPs, the resulting nanocomposite with Lup-2gβCD-AgNPs, which indicates the formation of the silver crystal structure. X-ray peaks in a wide range of angles of 2θ (30° < 2θ < 80°) showed that peaks at 38.02°, 44.07°, 64.35° and 77.21° can be attributed to 111, 200, 220 and 311 crystalline structures of a face-centered cubic (fcc) silver nanocrystal, respectively (Ag XRD Ref. No. 00-004-0783) [16].

The intensity of the peaks and their clarity indicate that Lup-2gβCD-AgNPs have a highly crystalline nature. It was also found that the intensities of 111, 200, 220, and 311 reflections due to the Ag-NPs phase increase along with an increase in AgNPs in the studied media. No other peaks present as impurities were found on the X-ray images. Thus, these results provide clear evidence of the presence of AgNPs in the Lup-2gβCD-AgNPs composition. 

**Figure 4:**
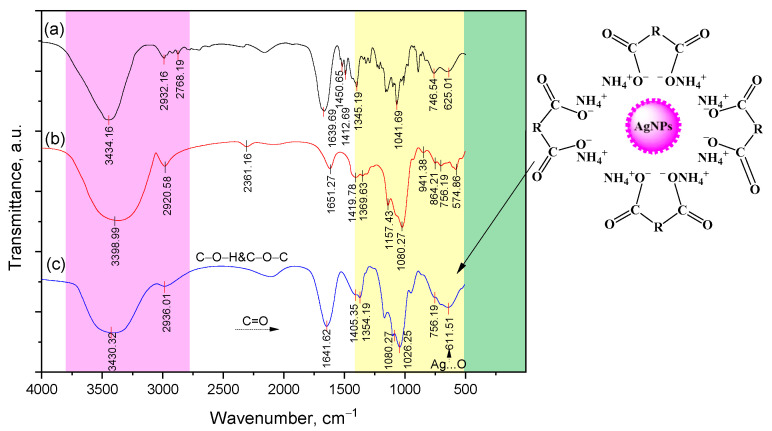
(a)-IR Fourier spectra of Lup; (b)-β-CD; (c)-Lup-2gβCD-AgNPs.

The emission of a clathrate complex of Lup inclusions with 2gβCD-AgNPs was also confirmed by FT-IR spectra (Figure 4). The FT-IR spectrum of the clathrate complex Lup-βCD-AgNPs revealed slight shifts in the characteristic absorption bands of 2gβ-CD functional groups. These shifts indicate the absence of covalent interactions between Lup and the internal functional groups of β-CD. A wide oscillation band in the range from 3425 to 3223 cm^−1^ indicates the deprotonation of OH groups of β-CD in an alkaline solution, which facilitates the synthesis and stabilization of AgNPs [30]. In the area of 1641 cm^−1^ of the product (c), there is an intense band characteristic of the C=O group of carboxylic acid. The bands caused by CO fluctuations merged into a wide envelope band at 1369–1354 cm^−1^. In the region of 1026 cm^−1^, the Lup-2gβCD-AgNPs nanocompositions exhibit a new wide absorption band of C-O-C groups. Similar data are typical for the Lac-2gβ-CD-AgNPs inclusion complex.

The structural features of the Lup (Lac) inclusion complexes and its β-CD inclusion complexes were also previously characterized by ^1^H-, ^13^C-NMR, spectroscopy, and two-dimensional analysis of COSY (^1^H-^1^H) and HMQC (^1^H-^13^C) spectra. Analysis of these spectra showed that the greatest difference in the values of chemical shifts of protons in the Lup-βCD inclusion complex is observed in H-3 (0.11 ppm) and H-5 (0.14 ppm) atoms. The formation of the 2gβCD-AgNPs, Lup (Lac)-2gβCD and Lup (Lac)-2gβCD-AgNPs nanocomposition was also studied using ^1^H-, ^13^C-NMR and COSY, HMQC, HMBC spectroscopy Figures S1a–d and S2a–e. It should be noted that the nature of the spectrum of the complex is influenced by the shielding effect of the interaction of AgNPs with 2gβCD. The FT-IR spectra of the Lup (Lac)-2gβCD clathrate complexes (2:1) revealed slight shifts in the characteristic absorption bands of the 2gβCD functional groups. This observation indicates the absence of covalent interactions between Lup and the internal functional groups of 2gβCD Figure S3a–e.

### 2.2. Thermogravimetric Analysis of 2gßCD-Lup(Lac)-AgNPs

#### 2.2.1. Thermal Decomposition of Lup and Its Derivatives

**Figure 5:**
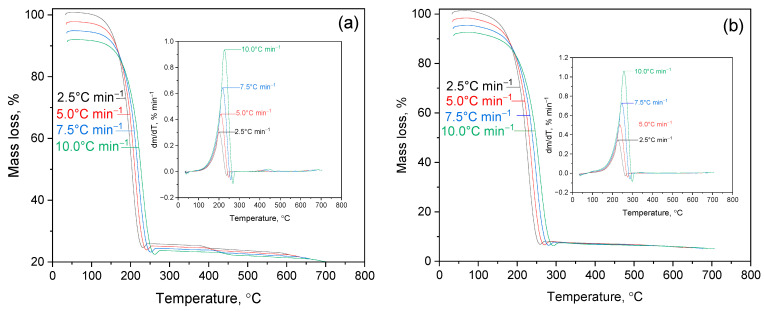

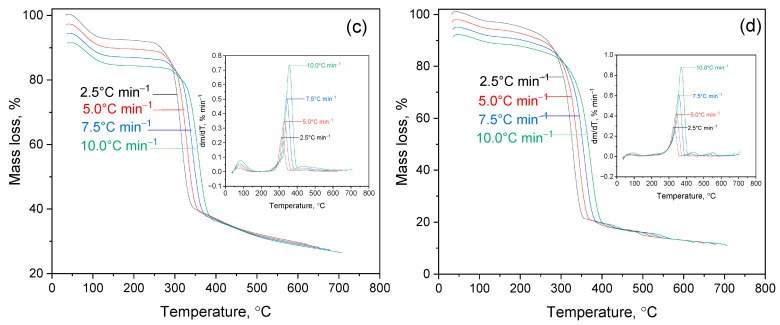
Thermogravimetric (TG) and differential thermogravimetric (DTG) curves of thermal decomposition of the following samples: (**a**)—Lup; (**b**)—Lac; (**c**)—Lup-2gβCD and Lup-2gβCD-AgNPs; (**d**)—Lup-2gβCD and Lup-2gβCD-AgNPs/acetone.

The thermal decomposition of Lup and its derivatives were studied by thermogravimetric analysis (TGA) in an inert gas atmosphere. Temperature gradients of 2.5, 5.0, 7.5, and 10.0 °C/min were used to evaluate the effect of the heating rate on thermal stability and decomposition characteristics. Figure S4 shows the proposed mechanisms of thermal decomposition: (a)—Lup; (b)—Lac; (c)—Lup-2gβCD-AgNPs. Figure S5 shows a block diagram of the stages of kinetic analysis using the method of model-free kinetics (NPK). The diagram illustrates the main stages of data processing. These include matrix formation, singular value decomposition (SVD), determination of the functional dependence R = g(α)·h(T), interpolation, construction of a 3D surface, and subsequent derivation of the kinetic model. Stepwise breaking of molecular bonds results in the formation of light volatile compounds and intermediate residues. In option, this process is altered due to the catalytic activity of silver nanoparticles (Figure S5). Figure 5 shows TG and differential thermogravimetric (DTG) curves for the following samples: (a)—Lup (plant alkaloid); (b)—Lac; (c)—Lup inclusion complex with 2gβCD and silver nanoparticles (Lup-2gβCD-AgNPs); (d)—similar a complex obtained in an acetone medium (Lup-2gβCD-AgNPs/acetone).

In the temperature range up to 100 °C, an initial loss of mass is observed, associated with the removal of adsorbed moisture and volatile components from the surface of the samples. In the region of 200–400 °C, an intensive decrease in mass occurs due to the destruction of the alkaloid structure and the release of low-molecular products. The DTG curves indicate that the decomposition process occurs in multiple stages. An increase in the heating rate causes the degradation peaks to shift toward higher temperatures, which reflects the kinetic nature of these processes.

(a) Lup (native compound)/TG curves demonstrate lower heat resistance compared to Lac; the onset of decomposition is observed at a lower temperature (~200–300 °C). DTG curves have more pronounced peaks at low temperatures, which indicates early destruction of the Lup structure.

(b) Lac. The TG curves exhibit a single major stage of mass loss within the temperature range of 200–350 °C, indicating thermal decomposition of the Lac molecule. The DTG curves show one intense peak at a maximum decomposition temperature of 260 °C (T_max_ = 260 °C). This peak shifts toward higher temperatures as the heating rate increases, which is a typical feature of kinetically controlled processes.

(c) The Lup-2gβCD-AgNPs complex. The TG curves show the complex nature of the decomposition, possibly due to the interaction between the components of the included complex. DTG curves: several peaks of 100 °C and 340 °C (T_max_) are present, indicating a gradual decomposition, degradation of β-CD, destruction of Lup, and possible involvement of silver nanoparticles in the catalytic activation of processes.

(d) The Lup-2gβCD-AgNPs/acetone complex. On the TG curves, flatter areas and a wide temperature range indicate multiple stages of decomposition. The DTG curves exhibit broader and smoother peaks compared to those in case (c). This behavior may result from the influence of residual acetone, which could stabilize intermediate products or alter the degradation pathway.

A comparison of the TG and DTG curves shows that modification of Lup by an acetyl group and its incorporation into a complex with 2gβCD and AgNPs leads to a change in the thermodestructive behavior of the compounds. Lac has a shift in the maximum decomposition rate (T_max_) to higher temperatures, which indicates an increase in thermal stability. In the case of the complexes, decomposition occurs in multiple stages, including the degradation of 2gβCD and interactions with AgNPs. This behavior indicates a catalytic effect of the silver nanoparticles and suggests alterations in the kinetic pathways of pyrolysis.

TG curves (on the left) show mass loss as a function of temperature, and DTG curves (on the right) the rate of decomposition. The experiments were carried out at different heating rates: 2.5, 5.0, 7.5 and 10.0 °C/min in an inert gas atmosphere.

Thus, a comparison of the TG and DTG curves shows that the modification of Lup and the formation of complexes significantly change its thermal behavior. These changes reflect the effect of functionalization and complexation on the stability and nature of decomposition.

A kinetic analysis was performed to quantify the catalytic effect and determine the activation energies. In order to gain a deeper understanding of kinetic behavior and quantify the various stages of decomposition, modern kinetic analysis methods were used. In particular, the Shestak–Berggren model [31] and the non-parametric kinetics (NPK) technique [32] were used, which avoided the need for an a priori assumption about the reaction mechanism. The results of kinetic modeling are presented in the following sections.

Thus, a comparison of the TG and DTG curves shows that modification of Lup, both by acetylation and through the formation of inclusion complexes with 2gβCD and silver nanoparticles (2gβCD-AgNPs), significantly changes its thermal behavior. The thermogravimetric data obtained (TG/DTG) showed a shift in the temperature of the maximum decomposition rate (T_max_) with chemical modification, which indicates a change in the thermal stability of the compounds. These changes reflect the influence of functionalization and complexation on the stability and nature of destructive processes. From a practical perspective, the observed differences in thermal stability and decomposition rates can be leveraged to intentionally control the release kinetics of active substances. This approach is particularly relevant for thermosensitive pharmaceutical formulations and agrochemical delivery systems.

#### 2.2.2. Kinetic Analysis of Thermal Decomposition of Lup Derivatives

To evaluate the kinetics of thermal decomposition of Lup derivatives, isothermal data were used, processed using model-free methods: the Friedman differential method [33] and the Ozawa–Flynn–Wall integral method (OFW) [34]. These methods make it possible to determine the dependence of the apparent activation energy (*E_a_*) on the degree of transformation (α), without assuming a specific reaction mechanism or the type of kinetic function g(α). The Friedman method [33] uses a logarithmic relationship between the conversion rate *da*/*dt* and the return temperature 1/T for a given value α, which allows us to graphically determine the activation energy along the slope of a straight line. The value of the preexponential multiplier A is usually estimated based on the Arrhenius equation assuming a first–order reaction, g(α) = (1 – α), averaged over all heating rates.

The Ozawa–Flynn–Wall method [34] is represented by an integral equation that makes it possible to construct the dependence of ln(β) on 1/T without the need to know the specific form of g(α). The slope of the obtained lines corresponds to −1.052Ea/R.

**Figure 6:**
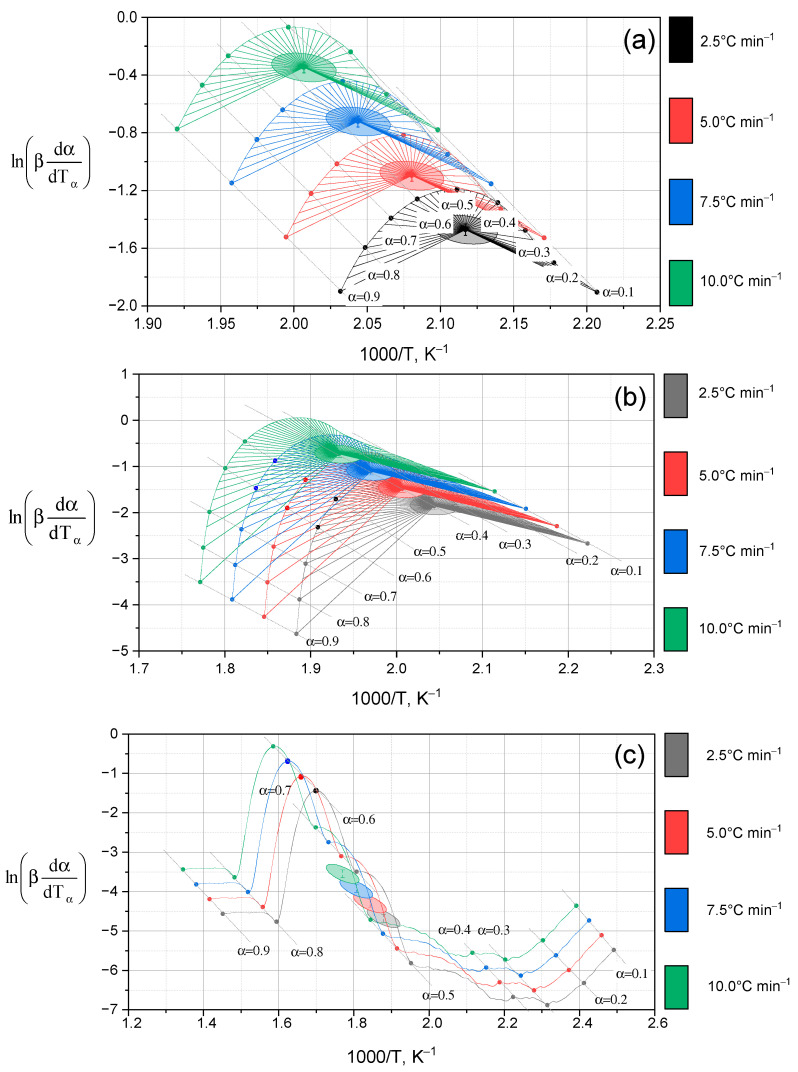

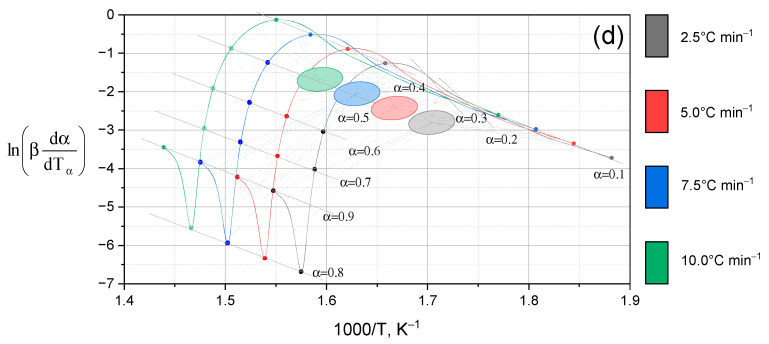
Dependences of the logarithm of the derivative α on the inverse temperature constructed for different degrees of transformation (α = 0.1, … 0.5, … 1.0) at four heating rates (β = 2.5, 5.0, 7.5, 10.0 °C min^−1^): (**a**)—Lup; (**b**)—Lac; (**c**)—Lup-2gβCD-AgNPs; (**d**)—Lup-2gβCD-AgNPs/acetone inclusion complex.

Graphical processing of experimental data by the Friedman method [33] was performed for all the studied compounds: Lup, Lac, a complex with 2gβCD and silver nanoparticles (Lup (Lac)-2gβCD–AgNPs), as well as a similar complex in an acetone medium. The dependences of the logarithm of the derivative α on the inverse temperature were constructed for different degrees of transformation (α = 0.1, … 0.5, … 1.0) at four heating rates (β = 2.5, 5.0, 7.5, 10.0 °C^.^ min^−1^) (Figure 6).

As can be seen from Figure 6, the activation energy of *E_a_* significantly depends on the degree of transformation of α, which reflects a change in the reaction mechanism at various stages of decomposition. In the early stages (α from 0.1 to 0.4), *E_a_* is relatively low, which may be due to the destruction of easily activated regions of the molecule. As the conversion degree (α) increases, the activation energy also rises. This trend is attributed to the formation of more stable fragments and residual structures that require higher energy for decomposition. This behavior is confirmed for all compounds, but the lowest *E_a_* values are observed for complexes with AgNPs, which indicates a possible catalytic activity of silver nanoparticles.

For a more detailed analysis of the mechanism of thermal degradation of Lup derivatives, the activation energy (*E_a_*) was estimated using model-free approaches: the Friedman differential method [33] and the Ozawa–Flynn–Wall integral method (OFW) [34]. The obtained *E_a_* values at various degrees of transformation (α = 0.1–0.9) are shown in Table 1. All calculations were performed based on data obtained at four heating rates (β = 2.5; 5.0; 7.5; 10.0 °C min^−1^), while the standard deviation was estimated based on the results of three parallel measurements.

In the case of Lup (a), the value of the activation energy shows a moderate decrease with an increase in the degree of transformation, which indicates a single-stage decomposition. The energy profile obtained by the Friedman method [33] varies from 88.35 ± 1.20 to 83.89 ± 1.32 kJ mol^−1^, whereas according to the OFW method it ranges from 90.26 ± 1.00 to 81.52 ± 1.21 kJ mol^−1^. This dynamic reflects a decrease in the availability of reacting centers as decomposition progresses. Lac (b) is characterized by similar *E_a_* values, but there is a slight decrease in the initial energy barrier. This may be due to the presence of an acetyl group, which increases the mobility of the molecule and facilitates the initial stages of decomposition. The maximum values are reached at α = 0.1–0.3. Then the parameters stabilize in the range of 83.5–85 kJ mol^−1^.

The most complex behavior is observed in the Lup-2gβCD-AgNPs (c) complex. The activation energy at the initial stages (α = 0.1) reaches ~95 kJ mol^−1^, then decreases significantly at α = 0.3–0.5, which may indicate a gradual degradation of the complex components-first 2gβCD, then Lup. The contribution of silver nanoparticles probably determines the growth of *E_a_* in the final stages, confirming the presence of catalytic involvement. For the Lup-2gβCD-AgNPs/acetone (d) complex, the energy parameters exhibit the smallest α fluctuations, remaining in the range of 83-87 kJ mol^−1^. This may indicate the stabilizing role of the acetone solution in the formation of the complex, as well as the uniform course of decomposition without pronounced stages. Overall, comparison of all samples reveals that modification of the Lup molecule through acetylation and complexation with 2gβCD and AgNPs significantly influences the kinetic parameters. These modifications reduce the activation energy barriers and transform the degradation mechanism from a simple to a multi-stage process.

To assess the statistical significance of differences in activation energy (*E_a_*) values obtained from various kinetic methods, we employed the Friedman differential method [33], the Ozawa–Flynn–Wall integral method [34], the model-free approach (NPK) [32], and the Shestak–Berggren model method [31]. Additionally, a one-way analysis of variance (ANOVA) and paired Student’s *t*-tests were conducted (Table 2). The analysis was carried out separately for each of the studied samples: Lup, Lac, and Lup inclusion complex with 2gβCD and silver nanoparticles (Lup-2gβCD-AgNPs), as well as a similar complex synthesized in an acetone medium (Lup-2gβCD-AgNPs/acetone). The purpose of the statistical analysis was to determine whether the observed differences in activation energy values are statistically significant or merely random. This assessment considered the effects of chemical modification, complexation, and catalytic components on the kinetics of thermal decomposition.

In particular, to compare the results obtained by the Friedman [33] and OFW [34] methods, a paired Student’s *t*-test was performed at a confidence level of 95% (*p* < 0.05). Statistical verification was carried out for each sample across different degrees of transformation (α = 0.1–0.9). For Lup (a), the differences between the methods were found to be statistically insignificant (*p* > 0.05) at all stages of conversion. This result indicates good agreement between the differential and integral approaches. A similar trend was observed for Lac (b), which confirms the reproducibility of calculations and the one-stage nature of its thermal degradation. However, for more complex systems such as Lup-2gβCD-AgNPs (c) and Lup-2gβCD-AgNPs/acetone (d), statistically significant differences (*p* < 0.05) between the calculation methods were found at higher α values (*p* ≥ 0.5). This may be due to the multi-stage nature of thermal degradation in these complexes. Another factor is the different sensitivity of kinetic methods to distributed reaction pathways. This is especially true when catalytically active silver nanoparticles are present. Thus, combining several kinetic approaches with statistical analysis improves calculation reliability. It also helps identify features of the decomposition mechanism of complex alkaloid derivatives. As part of the further study of the kinetic characteristics of the studied samples, the method of non-parametric kinetics (NPK) was applied [31,32]. This approach allows analysis of reaction parameters without prior assumptions about the mechanism. This feature makes it especially effective for studying complex, multi-stage processes. This paper presents a flowchart illustrating the sequence of processing experimental data using the NPK technique [32]. The method is characterized by high accuracy and analytical detail. It provides a deep understanding of the thermal decomposition of substances, including bond breaking, phase transformations, and structural rearrangements. These processes are typical for the pyrolysis of organic compounds and their derivatives, such as Lup derivatives.

**Figure 7:**
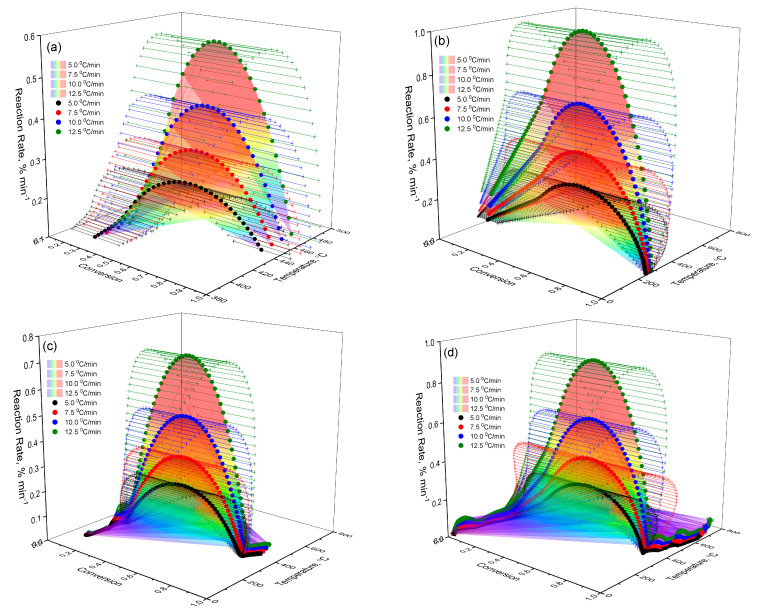
Three-dimensional dependences of the reaction rate (*da*/*dT*) on temperature (*T*) and degree of transformation (α) for the studied samples: (**a**)—Lup; (**b**)—Lac; (**c**)—Lup-2gβCD-AgNPs inclusion complex; (**d**)—a similar complex synthesized in an acetone medium (Lup-2gβCD-AgNPs /acetone).

In order to better understand the thermokinetic behavior of the studied samples, the dependence of the decomposition rate (*da*/*dT*) on temperature (*T*) and degree of transformation (α) was visualized in the form of three-dimensional graphs (Figure 7). These surfaces allow tracing the features of thermal decomposition for each substance. This includes differences arising from structural modifications and the effects of complexation [33,34,35,36,37]. Below is a step-by-step description of the thermokinetic characteristics of each sample:

(a) Lup. As follows from the data in Figure 7a, when heating Lup, a typical increase in the reaction rate is observed with increasing temperature, reaching a maximum at the degree of transformation a_max_ ≈ 0.5. After reaching this limit, the reaction rate begins to decrease. This decrease is associated with the depletion of active components and the formation of thermodynamically stable intermediates. The surface geometry is oblique and clearly reflects a kinetically controlled regime. In this regime, the reaction is most intense during the initial phase but slows down significantly in the later stages of the transformation.
(b) Lac. According to Figure 7b, the general nature of the sample surface demonstrates a typical kinetic dependence: with increasing temperature, the reaction rate increases to a certain limit, after which it begins to decrease as the reagent is depleted. The reaction rate peaks are reached in the range of conversion degrees a_max_ ≈0.6, which indicates the active stage of the destruction of the Lac molecule. A comparison of the curves at different heating rates (β) shows that increasing the heating rate causes the reaction peaks to shift toward higher temperatures. This shift is a characteristic feature of kinetically controlled processes. The graph surface for Lac exhibits a more pronounced maximum and steeper gradients compared to native Lup. This indicates more intense thermal decomposition and likely lower heat resistance, which may be attributed to the presence of an acetyl group.
(c) Lup inclusion complex with 2gβCD and silver nanoparticles (Lup-2gβ-CD-AgNPs). The surface of Figure 7c is characterized by a more complex shape, including several local maxima, which indicates the multi-stage decomposition process of the complex. The main maximum of the reaction rate is observed in the range α = 0.4–0.6 and a temperature of about 270–300 °C, which indicates the active destruction of both Lup and 2gβCD. The peak shift with increasing β indicates the kinetic influence of temperature and is typical for reactions involving several components. Comparison with Figure 7a,b shows that, in the case of the Lup-2gβCD-AgNPs inclusion complex, the reaction proceeds at a higher decomposition rate. This increase may be attributed to the catalytic effect of silver nanoparticles. Additionally, changes in thermal stability due to the formation of the inclusion complex could contribute to this behavior. Steeper alpha responses may also indicate a sharper transition between stages, characteristic of cooperative effects in complex compounds.
(d) Lup-2gβCD-AgNPs/acetone (inclusion complex obtained in an acetone solution). The three-dimensional surface shown in Figure 7d demonstrates the most pronounced characteristics among all the studied samples. Several intense zones of increased reaction rate are observed. This observation indicates the multi-stage nature of the thermal decomposition of the complex. The main peak of the reaction is located in the temperature range of 260–310 °C and corresponds to the degrees of transformation α ≈ 0.5–0.7.

The NPK method [32] demonstrated that Lup-2gβCD-AgNPs inclusion complexes exhibit reduced energy barriers and increased reactivity. These properties make them promising candidates for use in thermosensitive functional materials. Unlike the previous samples, surface (d) exhibits gentler gradients in the high-temperature range and a gradual decrease in the reaction rate. This behavior may be attributed to the influence of the acetone solution on the stability of the complex. The presence of acetone during synthesis likely contributes to improved distribution of silver nanoparticles. This, in turn, enhances the catalytic effect and lowers the reaction’s energy barrier. Visually, the surface appears highly uniform, with no sudden jumps. This suggests that the reactions proceed in an orderly manner. The increased reaction rates observed over a wider range of conversion degrees (α) may indicate a synergistic effect among 2gβCD, AgNPs, and residual acetone molecules. These components presumably stabilize transition states and help lower the energy barrier of the reactions. Consequently, the Lup-2gβCD-AgNPs/acetone complex exhibits the highest reactivity, stability, and pronounced kinetic selectivity during thermal degradation. This makes it a promising candidate for targeted thermocatalytic control.

The kinetic profiles of thermal decomposition show the potential of modified compounds. This is especially true for those containing AgNPs and synthesized in acetone. These materials can be used in temperature-controlled delivery and precision thermal synthesis. Although the model-free approach is informative, we used the Shestak–Berggren model [31]. This model works well for processes with variable kinetics. It helped quantify the mechanism and define the kinetic function’s shape. Using it, the reaction profile with parameters *n* and *m* was described. The fit of the experimental data to a specific decomposition model was also evaluated.

**Figure 8:**
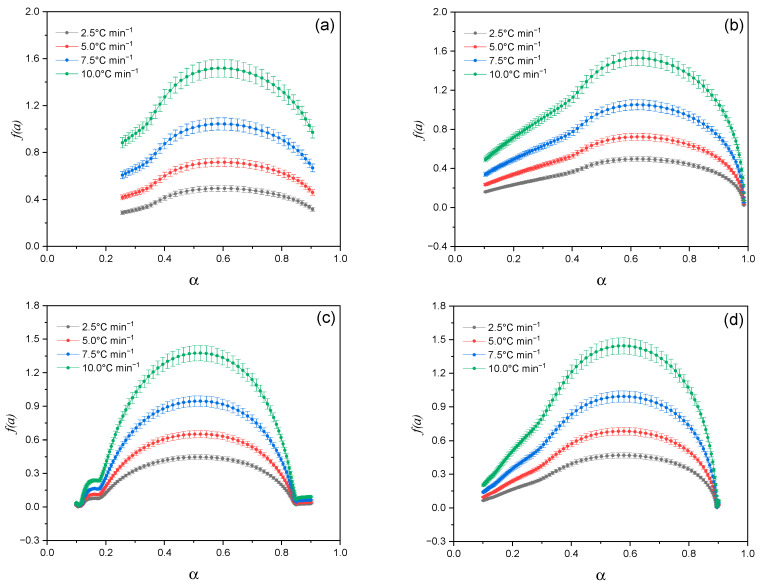
Dependences of the reaction rate (*da*/*dT*) on the degree of transformation (α) at different heating rates (β = 2.5, 5.0, 7.5 and 10.0 °C min^−1^) for the studied samples: (**a**)—Lup; (**b**)—Lac; (**c**)—inclusion complex Lup-2gβCD-AgNPs; (**d**)—Lup-2gβCD-AgNPs inclusion complex in acetone solution.

As can be seen from Figure 8, all samples exhibit a typical kinetic dependence. The reaction rate increases as the degree of transformation (α) rises, reaching a maximum value (α_max_). After this point, the reaction rate begins to decline.

This profile is typical for reactions limited by the amount of available reagent and product stabilization. Pronounced maxima in the range of α ≈ 0.4–0.6 are observed for Lup (a) and Lac (b), while Lac shows a sharper peak, which indicates its lower thermal stability. Complexes (c) and (d) exhibit higher values of the *da*/*dT* velocity and a shift of peaks to the region of higher α with increasing β, which may indicate the catalytic effect of AgNPs and a change in thermokinetic behavior in the presence of 2gβCD and acetone. This shape of the curves is consistent with the 3D visualization data (Figure 7), confirming the multi-stage mechanism of thermal decomposition and kinetic selectivity of the modified systems.

Two independent approaches were used to verify the kinetic parameters of the thermal decomposition of Lup derivatives: the model-free nonlinear regression method non-parametric kinetics (NPK) and the Shestak–Berggren model [31,32]. The results of calculations of the average activation energy (*E_a_*) and the pre-exponential multiplier ($\bar{A}$) are shown in Table 3. Empirical parameters (*m*, *n*) for the reaction function of the form *a^m^*(1 *− α*)*^n^* used in the Shestak–Berggren [31] are also indicated. The analysis shows a high degree of consistency between the activation energy estimates obtained by the NPK and Shestak–Berggren [31] methods, which confirms the reliability of the results. In all cases, the average activation energy is in the range of 84.0–86.0 kJ mol^−1^, with the highest value recorded for the Lup-2gβCD-AgNPs complex, which may indicate a complex multi-stage degradation involving all components of the complex. The parameters *m* and *n*, characterizing the shape of the kinetic function, indicate a deviation from the classical first-order scheme. For the modified forms (b–d), the value of *n* is less than 0.5. This suggests that autocatalytic processes influence the reaction rate. The Lup-2gβCD-AgNPs (c) complex exhibits the highest values of $\bar{A}$ and *m*/*n* parameters, which may be related to catalysis by silver nanoparticles. Thus, the two independent methods, NPK [32] and Shestak–Berggren [31], allow evaluation of reaction energy barriers. Additionally, it provides deeper insight into the decomposition mechanism, especially in complex systems.

Statistical data processing was performed to quantify the consistency between the results obtained using the model-free NPK method [32] and the Shestak–Berggren model approach [31]. The activation energy (*E_a_*) values calculated by two methods for four studied samples (Lup, Lac, Lup-2gβCD-AgNPs, Lup-2gβCD-AgNPs/acetone) were compared using the Student’s paired *t*-test and univariate analysis of variance (ANOVA) at a significance level of *p* < 0.05.

The results of the *t*-test showed no statistically significant differences between the average *E_a_* values for all studied samples (*p* > 0.05), including Lup (*p* = 0.231), Lac (*p* = 0.151), the Lup-2gβCD-AgNPs complex (*p* = 0.770) and Lup-2gβCD-AgNPs/acetone (*p* = 0.239). This indicates a high degree of agreement between the two calculation methods. This is despite differences in their approaches. The Shestak–Berggren model method [31,32] assumes a specific form of the transformation function. In contrast, the NPK method does not require a priori assumptions. ANOVA analysis also confirmed the absence of significant differences (*F* = 0.0263, *p* = 0.872). This shows that variations in activation energy estimates between the methods fall within random fluctuations and are not statistically significant. Therefore, it can be concluded that the NPK and Shestak–Berggren methods [31,32] provide comparable results for calculating kinetic parameters. Their combined use enhances the reliability of interpreting thermogravimetric data. This is especially important when analyzing complex substances, such as Lup derivatives.

### 2.3. Results of the Study of Cytotoxic, Antibacterial and Analgesic Activity of New Lup Derivatives

#### 2.3.1. Antimicrobial Activity of the Samples

The results of the study of the antimicrobial activity of four samples by serial dilution are shown in Table S1. Antimicrobial testing showed that the samples exhibit activity against Gram-positive strains *Staphylococcus aureus* and *Bacillus subtilis*, as well as the Gram-negative *Escherichia coli.* Their minimum inhibitory concentrations (MICs) ranged from 12.5 to 50 mcg/mL. Among all tested samples, the compound Lup-2gβCD demonstrated the strongest antibacterial activity against the Gram-positive strain *Bacillus subtilis* ATCC 6633. Its MIC was 6.3 mcg/mL. The Lup-2gβCD compound also showed a moderately pronounced antibacterial effect against the Gram-positive test strain *Staphylococcus aureus* and a moderate antifungal effect against the fungus *Candida albicans* (MIC = 25 mcg/mL). The Lac sample showed moderate antibacterial activity against Gram-positive test strains *Staphylococcus aureus* ATCC 6538 and *Bacillus subtilis* ATCC 6633. Their MIC were 12.5 and 25 mcg/mL, respectively. The compounds Lup-2gβCD-AgNPs and Lup showed moderate antibacterial activity only against the Gram-positive strain *Staphylococcus aureus* ATCC 653, with a MIC of 25 mcg/mL. Against the other test strains, these substances exhibited weak antibacterial activity, with a MIC of 50 mcg/mL (Table S1).

#### 2.3.2. Cytotoxic Activity of the Samples

The synthesized new compounds were tested for lethality by *Artemia salina* (Leach). Cytotoxic activity based on the percentage of larval death was assessed after 24 h exposure to the drugs. According to Meyer et al. [37], who classified the substances into toxic (LC_50_ value < 1000 mcg/mL) and non-toxic (LC_50_ value > 1000 mcg/mL), almost all the tested compounds showed good cytotoxic activity of artemia compared with the control compound. The cytotoxicity of the compounds was evaluated in a test for the survival of larvae of the crustacean *Artemia salina* (Leach) under in vitro cultivation conditions [37,38]. It was found that the samples-Lup, Lac, Lup-2gβCD, Lup-2gβCD-AgNPs exhibit cytotoxic activity against larvae of marine crustaceans *Artemia salina* (Leach). The cytotoxic activity of the Lup-2gβCD-AgNPs samples is more pronounced (LD_50_ 54.3 mcg/mL) than that of the other samples presented (the reference drug is dactinomycin (50 mg, USP 1162400) (Table S2).

#### 2.3.3. Results of the Study of the Analgesic Activity of the Samples

The study found that a sample of Lup acetate at a dose of 25 mg/kg exhibited pronounced analgesic activity in a chemical irritation model of the peritoneum. It significantly reduced the number of acetic cramps in rats by 57.5% compared to the control (Table S3). The analgesic activity of this compound Lup acetate is 4.5% higher than the analgesic activity of the comparison drug is diclofenac sodium. The Lup-2gβCD-AgNPs sample demonstrated moderate analgesic activity in the acetic cramps test. It reduced the visceral nociceptive response to acetic acid by 42.4% compared to the control. Samples of Lup and Lup-2gβCD at doses of 25 mg/kg showed weak analgesic activity in the same model. They reduced the number of acetic cramps by 29.4% and 18.6%, respectively, compared to the control (Table S3).

## 3. Materials and Methods

### 3.1. Determination of the Structural Characteristics of the Lup(Lac)-2gβCD-AgNPs Nanocomposite

The following reagents were used:

2-Hydroxypropyl-β-Cyclodextrin (2gβCD) (99.5%) (molar mass-1541.6 g/mol, m.p. 299 °C) with dilution white crystalline substance. 2gβCD was obtained from Wacker Chemie (Munich, Germany). Lup (m.p. 69–71 °C (EtOH, [α]_D_ 30.5 (s 0.41, MeOH) (m.p. 68–69 °C (EtOH), [α]_D_ 23.5). All other materials were purchased from Sigma Aldrich (Dublin, Ireland) and were used without further purification. All solutions were prepared in Elga Millipore deionized water. Silver nitrate (AgNO_3_) and NaOH hydroxide were purchased from Sinopharm Chemical Reagent Co., Ltd. (Shanghai, China). All solutions were freshly prepared using double distilled water and stored in the dark to avoid any photochemical reactions. All glassware used in the experimental procedures was cleaned with a fresh HNO_3_/HCl solution (3:1 by volume), thoroughly rinsed with double distilled water, and dried before use.

The absorption spectra of Lup-2gβCD-AgNPs and Lac-2gβCD-AgNPs in the ultraviolet range were recorded using an N60 Implen UV-visible spectrophotometer. Samples of substances for analyzing spectra in the ultraviolet range were prepared by mixing 1 mL of solution with 10 mL of water. ^1^H-, ^13^C-NMR spectra were obtained using a Bruker Avance 600M NMR instrument using D_2_O as a solvent.

The infrared spectrum was measured using an external Nicolet iS50 Fourier spectrometer (KBr). A total of 36 scans were acquired, ranging from 4000 to 400 cm^−1^ at a resolution of 4 cm^−1^. The X-ray diffraction pattern was measured on an XD6 X-ray diffractometer at 40 kV and 30 mA with a scanning speed of 5° per minute and a scanning range of 20°–90° using Cu Ka radiation (l = 0.1546 nm).

TEM was performed on a JEM-1400 transmission electron microscope operating at an accelerating voltage of 80 kV. TEM samples were prepared by placing several drops of freshly prepared sol onto a copper grid coated with a carbon support film. The samples were then dried at room temperature. The particle size distribution was obtained using a TEM image and ImageJ software (1.53t).

### 3.2. Study of the Thermal Behavior and Kinetics of Decomposition of Lup and Its Modified Form

Thermal decomposition of Lup and its derivatives (Lup-2gβCD-AgNPs, Lup-2gβCD-AgNPs/acetone, and Lac-2gβCD-AgNPs) was studied using a LabsysEvo TG-DTA/DSC thermal analyzer (SETARAM, Caluire-et-Cuire, France). The analysis was carried out in corundum crucibles over a temperature range of 30 to 1000 °C. All measurements were conducted under a nitrogen atmosphere. The consumption of protective and purge gas was 20 and 50 mL/min, respectively. The heating rates were 2.5, 5.0, 7.5 and 10.0 °C/min. The mass of each test sample ranged from 10.0 ± 0.5mg. Each measurement was performed in triplicate to ensure reproducibility and allow for subsequent statistical averaging. Experimental data processing and graph visualization were performed using the OriginPro 9.0 and Anaconda software packages (Python 3.10) with the NumPy, SciPy, and Matplotlib libraries (3.10.1).

The model-free Friedman [33], Ozawa–Flynn–Wall [34], and non-parametric kinetics (NPK) methods [32] were additionally used to estimate kinetic parameters. The model analysis was carried out using the Shestak–Berggren equation [31], which allowed for a detailed comparison of the kinetic modes of thermal degradation. To verify the statistical significance of the differences between the methods, the Student’s paired *t*-test and univariate analysis of variance (ANOVA) were applied at a significance level of *p* < 0.05 based on calculations for each α.

### 3.3. Investigation of Biological Properties of Lup Derivatives

#### 3.3.1. Antibacterial Activity of the Samples (In Vitro)

Antibacterial activities of capped and uncapped AgNPs of different silver concentrations were determined by a microtiter well method [35]. The negative control consisted of AgNPs (100 μL) added to sterile Mueller–Hinton agar (Lab M) and stored at 4 °C. Samples were doubly diluted in water (100 CFU/mL) in microtiter wells and bacteria (100 μL; 10^6^ CFU/mL) were added. Negative (AgNPs 100 μL + sterile MHB 100 μL) and positive (sterile MHB 100 μL + bacterial suspension 100 μL) control wells and a sterility control blank (sterile MHB 200 μL) were included in each assay. Plates were incubated (18 h) in a microtiter plate reader (PowerWave tm Microplate spectrophotometer; BioTek, Winooski, VT, USA) at 36.0 °C. Samples were tested in duplicate on each plate and each plate was analyzed in triplicate.

The antibacterial activity of the samples was evaluated using the serial dilution method. Test organisms included Gram-positive *Staphylococcus aureus* ATCC 6538 and *Bacillus subtilis* ATCC 6633; Gram-negative *Escherichia coli* ATCC 25922 and *Pseudomonas aeruginosa* ATCC 27853; and the fungus *Candida albicans* ATCC 10231. The minimum inhibitory concentration (MIC) was determined for each strain, following established protocols [35,36]. Test strains microorganisms used in this study were obtained from the American Collection of Standard Crops. The antibacterial drug ceftriaxone and the antifungal drug nystatin were used as comparison drugs and served as a positive control.

Minimum inhibitory concentration (MIC) was determined by sequential dilution of ethanol solutions of the studied samples in a nutrient broth. Suspensions of test strains at a concentration of 10^6^ CFU/mL were applied for the sequential dilution method. A suspension of test strains of microorganisms was prepared from daily cultures grown on mown agar at 37 °C for 24 h, for the fungus *Candida albicans*—at 30 °C for 48 h. The antimicrobial activity of the samples was studied at dilutions in the range of 1.56–50 micrograms/mL. A microbial suspension of 0.1 mL at a concentration of 10^6^ CFU/mL was added to each tube with a working dilution of each test sample. The procedure was repeated for all the cultures studied. A suspension of microbes with a nutrient medium without a sample was placed in control tubes (negative control). The mixture was incubated in a thermostat for 24–48 h, depending on the class of microorganism. The presence of turbidity in each tube was visually assessed. The tube containing a clear suspension and the lowest concentration of the antimicrobial agent was identified. This concentration corresponded to the MIC. The results were averaged over the data from three experiments.

#### 3.3.2. Cytotoxic Activity

The cytotoxicity of the synthesized compounds against *Artemia salina* (Leach) was evaluated in accordance with the methodology proposed by Meyer et al. [37,38]. The cytotoxicity of the samples was evaluated in the survival test of *Artemia salina* crustacean larvae. The experiments were carried out on larvae at the age of 2 days under in vitro cultivation conditions. The larvae were grown by immersing eggs of *Artemia salina* (Leach) crustaceans in artificial seawater and incubating for 48 h at a temperature of 37 °C. The sample was dissolved in 2 mL of ethanol, then 500 µL (3 parallels), 50 µL (3 parallels), 5 µL (3 parallels) were taken from this solution. After ethanol evaporation, 5 mL of artificial seawater was added to each bottle. Thus, if the initial weight of the sample was 2 mg, then the final concentrations in the sample were 100 mcg/mL, 10 mcg/mL and 1 mcg/mL, respectively, for each concentration in three repetitions. Ten larvae of two-day-old *Artemia salina* crustaceans were placed in each bottle with the image using a Pasteur pipette. After that, all vials were left at room temperature in the light for 24 h. After 24 h, the surviving and dead larvae were counted. Then, using the data obtained on the upper and lower limits of toxicity, the half toxic dose of the sample was calculated. Control-DMSO in equal amounts. The test was performed using ready-made samples, as well as a reference drug, dactinomycin (actinomycin D), which has antitumor (cytotoxic) activity (manufacturer Sigma Aldrich, St. Louis, MO, USA). The lethal concentrations of these compounds, leading to a 50% death rate of shrimp (LC_50_), and 95% confidence intervals were determined based on 24 h calculations using probit analysis and obtaining an LC_50_ value with a 95% confidence interval [37,38]. Statistical processing of the results was carried out using the FNI computer program.

#### 3.3.3. Analgesic Activity of the Samples

The analgesic activity of the tested compounds was evaluated using a chemical irritation model of the peritoneum. The model was based on intraperitoneal administration of acetic acid (Acetic Cramps test). The study was conducted on white outbred mice weighing 20–25 g [39]. Convulsions were caused by intraperitoneal injection of 0.75% aqueous solution of acetic acid at a dose of 1 mL per 100 g of animal body weight. The test substances were administered intragastrically 30 min before the administration of acetic acid. The number of convulsions was counted 20 min after intraperitoneal administration of acetic acid for 30 min. The studied substances in the form of starch mucus were injected intragastrically at a dose of 25 mg/kg using a special metal probe 30 min before the introduction of acetic acid. The control animals received an equi-volumetric amount of starch mucus. A decrease in the number of convulsions in animals, compared with the control group, served as an indicator of the analgesic activity of the studied substances. Diclofenac sodium at its effective dose of 8 mg/kg (ED_50_ = 8 mg/kg) was used as a comparison drug. Analgesic activity was expressed as a percentage decrease in the number of acetic cramps in the experimental rats compared with the control ones. The analgesic activity was calculated using the formula

% analgesic activity = ((*X_c_* − *X_exp_*)/*X_c_*) × 100%

where *X_c_* and *X_exp_* are the number of convulsions in the control and in the experiment, respectively.

### 3.4. Preparation of the Lac-2gßCD-AgNPs Inclusion Complex

**Figure 9:**
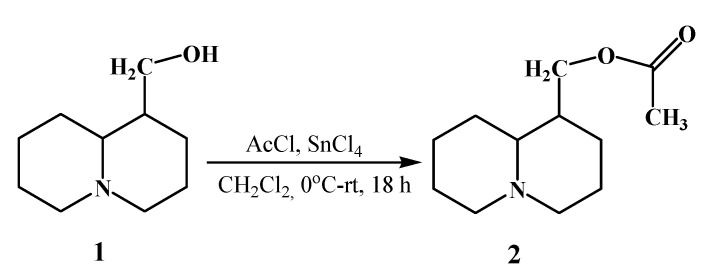
Synthesis of Lup acetate.

**Figure 10:**
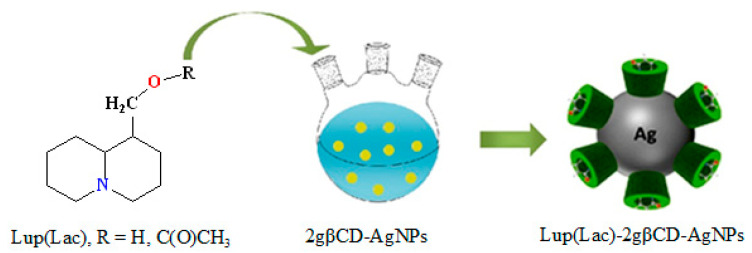
Schematic representation for the synthesis of Lup (Lac)-2gβCD-AgNPs.

The present study demonstrates the preparation and thermochemical characterization of an encapsulated cyclodextrin complex of Lup and Lac as an organic ligand in the 2gβCD-AgNPs nanocomposition. The modified composition of Lup (Lup)-2gβCD-AgNPs) and its acetate with nanosilver (Lac-2gβCD-AgNPs) can be considered a promising platform for improving stability and expanding its antibacterial biopotential. The synthesis of Lup acetate and its schematic representation for the synthesis of Lup (Lac)-2gβCD-AgNPs are shown in the following scheme (Figure 9 and Figure 10):

The synthesis of Lac was carried out by the interaction of Lup with acetyl chloride in the presence of SnCl_4_ (81% yield, Figure 9) according to the methodology [40]. We have previously published a study on the encapsulation mechanisms of the Lup molecule with cyclodextrin [41]. The synthesis of encapsulated Lup (Lac)-2gβCD-AgNPs compositions with nanosilver was obtained in two stages according to the following scheme: at the first stage, 2gβCD-AgNPs was synthesized by in situ reduction in accordance with the described method [16,17,18] with minor changes (Figure 10). The method described here at the first stage included the reduction of the [Ag(NH_3_)_2_]^+^ complex to metallic Ag^0^ with an aqueous solution of 2gβCD. 5 mL of 2gβCD solution (0.1 M) was added to 50 mL of water, to which NH_4_OH (10%) was gradually added during stirring to a pH value of 9.0. Then 1.5 mL of AgNO_3_ solution (0.001 M) was added dropwise to the resulting solution, and further reacted for 2 h at 85 ± 0.5 °C until a light yellow 2gβCD-AgNPs solution was obtained.

During further reaction, the color of the solution changed to an intense yellow-brown, indicating the formation of AgNPs nanoparticles. This may be due to the excitation of surface plasmon oscillations of AgNPs, which corresponds to the literature data [39]. An increase in the reaction time and the pH of the solution leads to the appearance of a dark brown, and then a gray color of the solution. At the next stage, the resulting 2gβCD-AgNPs solution was further used to encapsulate Lup and Lac molecules. Lup (Lac)-2gβCD-AgNPs complexes were obtained by slowly adding 5 mL of Lup (Lac) solution (0.01 M) to an aqueous solution of 2gβCD-AgNPs. Each experiment was repeated three times.

## 4. Conclusions

The alkaloid lupinine and its acetyl derivative attracted attention because of their bactericidal properties. These properties motivate the development of reliable methods for their biomedical application. In this study, the synthesis and properties of silver-functionalized 2-hydroxypropyl-β-cyclodextrin nanoparticles and their filling with lupinine and its acetyl derivative have been investigated. The obtained thermodynamic and kinetic data enable prediction of the stability of these clathrate complexes during long-term storage. They also help identify optimal conditions for complex formation. Encapsulated lupinine inclusion complexes were prepared. Its acetate derivative was combined with silver nanoparticles. This led to an expanded spectrum of antibacterial, cytotoxic, and antifungal activities.

## Figures and Tables

**Table 1:** Activation energy (*E_a_*, kJ mol^−1^) as a function of the degree of transformation (α), calculated by the Friedman and Ozawa–Flynn–Wall methods for the studied compounds.

Sample/*α*	Method	0.1	0.3	0.5	0.7	0.9
(a) Lup	Friedman	88.35 ± 1.20	85.75 ± 1.20	84.87 ± 1.12	84.40 ± 0.90	83.89 ± 1.32
	OFW	90.26 ± 1.00	87.77 ± 1.41	86.80 ± 1.13	85.3 ± 1.71	85.52 ± 1.21
(b) Lac	Friedman	86.36 ± 1.30	84.42 ± 1.23	83.90 ± 1.02	83.65 ± 1.54	83.49 ± 1.70
	OFW	88.61 ± 1.41	86.22 ± 1.31	85.68 ± 1.41	85.48 ± 1.90	85.01 ± 1.50
(c) Lup-2gβCD-AgNPs	Friedman	93.86 ± 1.10	83.98 ± 1.00	84.28 ± 1.20	84.64 ± 1.10	88.36 ± 1.00
	OFW	95.03 ± 1.51	85.85 ± 1.30	86.71 ± 1.20	86.18 ± 1.70	90.57 ± 2.00
(d) Lup-2gβCD-AgNPs/acetone	Friedman	86.37 ± 1.00	84.92 ± 1.10	84.59 ± 0.90	84.16 ± 1.00	83.43 ± 1.10
	OFW	88.98 ± 1.10	86.01 ± 1.21	86.68 ± 1.11	86.27 ± 1.30	85.03 ± 1.21

Note: All values are given as the mean ± standard deviation (*n* = 3).

**Table 2:** Statistical analysis of activation energy (E_a_) values of samples.

Composition	F-Statistic	*p*-Value (ANOVA)	*t*-Statistic	*p*-Value (*t*-Test)
(a) Lup	0.2839	0.6086	−1.0484	0.3536
(b) Lac	4.9978	0.0558	−15.6370	0.0001
(c) Lup-2gβCD-AgNPs	0.5119	0.4947	−8.1465	0.0012
(d) Lup-2gβCD-AgNPs/acetone	5.4098	0.0485	−7.3667	0.0018

**Table 3:** Calculated values of the activation energy and the pre-exponential multiplier for thermal decomposition of alkaloid derivatives.

Sample	${\bar{E}}_{NPK}$, kJ mol^−1^ ± SD	$\bar{A}$·10^7^, s^−1^ ± SD	Šesták–Berggren *α^m^*(1 *− α*)*^n^*		${\bar{E}}_{SB}$, kJ mol^−1^ ± SD	$\bar{A}$·10^7^, s^−1^ ± SD
			m	*n*		
(a) Lup	84.54 ± 0.12	1.45 ± 0.05	0.41	0.30	84.95 ± 0.12	2.22 ± 0.18
(b) Lac	84.01 ± 0.15	1.78 ± 0.04	0.35	0.20	84.24 ± 0.15	0.80 ± 0.10
(c) Lup-2gβCD-AgNPs	85.76 ± 0.75	2.54 ± 0.54	0.47	0.43	85.82 ± 1.50	3.98 ± 1.20
(d) Lup-2gβCD–AgNPs/acetone	84.87 ± 0.41	2.01 ± 0.05	0.42	0.30	84.63 ± 1.10	1.39 ± 0.75

Note: The activation energy (*E*_a_) is given in kJ.mol^−1^, and the pre-exponential multiplier (*Ā*) is in s^−1^. All values are presented as the average ± standard deviation based on the results of three independent measurements.

## Data Availability

The data that support the findings of this study are available within the article and the Supplementary Materials. Further data are available from the corresponding author upon reasonable request.

## Supplementary Materials

The following supporting information can be downloaded at https://www.mdpi.com/article/10.3390/molecules30163354/s1, Figure S1. ^1^H (a), ^13^C (b), COSY (^1^H-^1^H) (c) and HMQC (^1^H-^13^C) (d) NMR spectra of Lup; Figure S2. ^1^H (a), ^13^C (b), COSY (^1^H-^1^H) (c), HMQC (^1^H-^13^C) (d) and HMBC NMR spectra of lupacetat; Figure S3. ^1^H (a), ^13^C (b), COSY (c), HMQC (d), HMBC (e) NMR spectra of 2gβCD-AgNPs (D^2^O); Figure S4. The proposed mechanisms of thermal decomposition are (a)—Lup; (b)—acetylated Lup; (c)—Lup inclusion complex with β-CD and silver nanoparticles (Lup-2gβCD-AgNPs). Stepwise breaking of molecular bonds leads to the formation of light volatile compounds and intermediate residues. In option (c), the process is altered due to the catalytic activity of silver nanoparticles. Figure S5. A block diagram illustrates the main stages of kinetic analysis using the model-free kinetics method (NPK). These stages include matrix formation, singular value decomposition (SVD), determination of the dependence R = g(α)·h(T), interpolation, and construction of a 3D surface. The analysis is completed by deriving a kinetic model. Table S1 presents the antimicrobial activity of the samples. Table S2 contains data on their cytotoxic activity. Table S3 summarizes the analgesic activity of the samples.

## References

[1] Michael J.P. (2007). Indolizidine and quinolizidine alkaloids. Nat. Prod. Rep..

[2] Tasso B., Mattioli L.B., Tonelli M., Boido V., Chiarini A., Sparatore F., Budriesi R. (2023). Further quinolizidine derivatives as antiarrhythmic agents-3. Molecules.

[3] Takao K., Munakata R., Tadano K. (2005). Recent advances in natural product synthesis by using intramolecular Diels-Alder reactions. Chem. Rev..

[4] Nurmaganbetov Z.S., Nurkenov O.A., Khlebnikov A.I., Fazylov S.D., Seidakhmetova R.B., Tukhmetova Z.K., Takibayeva A.T., Khabdolda G., Rakhimberlinova Z.B., Kaldybayeva A.K. (2024). Antiviral Activity of (1*S*,9a*R*)-1-[(1,2,3-Triazol-1-yl)-methyl]-octahydro-1*H*-quinolizines from the Alkaloid Lupinine. Molecules.

[5] Magalhaes S., Fernandes F., Cabrita A.R.J., Fonseca A.J.M., Valentao P., Andrade P.B. (2016). Alkaloids in the valorization of European Lupinus spp. seeds crop. Ind. Crops Prod..

[6] Konrath E.L., Passos C.D.S., Klein-Junior L.C., Henriques A.T. (2013). Alkaloids as a source of potential anticholinesterase inhibitors for the treatment of Alzheimer’s disease. J. Pharm. Pharmacol..

[7] Tlegenov R.T., Dalimov D.N., Haitbaev H.H., Abduvahabov A.A., Uteniyazov K.U. (1990). Synthesis and antikholinesterase activites of a number of the alkaloids lupinine. Chem. Nat. Compound..

[8] Turdybekov K.M., Nurkenov O.A., Nurmaganbetov Z.S., Satpaeva Z.B., Turdybekov D.M., Makhmutova A.S., Fazylov S.D. (2020). Synthesis, crystal structure, and stability of N-lupinylphthalimide conformers. J. Struct. Chem..

[9] Frik K.M., Kamphuis L.G., Siddique K.M., Singh K.B., Foley R.C. (2017). Quinolizidine alkaloid Biosynthesis in Lupins and Prospects for Grain Quality Improvement. Front. Plant Sci..

[10] Gusarova N.K., Malysheva S.F., Oparina L.A., Belogorlova N.A., Tantsyrev A.P., Parshina L.N., Sukhov B.G., Tlegenov R.T., Trofimov B.A. (2009). Synthesis of novel alkaloid derivatives from vinyl ether of lupinine and PH-addends. Arkivoc.

[11] Schepetkin I.A., Nurmaganbetov Z.S., Fazylov S.D., Nurkenov O.A., Khlebnikov A.I., Seilkhanov T.M., Kishkentaeva A.S., Shults E.E., Quinn M.T. (2023). Inhibition of Acetylcholinesterase by Novel Lupinine Derivatives. Molecules.

[12] Su D., Wang X., Shao C., Xu J., Zhu R., Hu Y. (2011). Total Synthesis of (+)-Epilupinine via an intramolecular nitrile oxide-alkene cycloaddition. J. Org. Chem..

[13] Semenov V.E., Zueva I.V., Mukhamedyarov M.A., Lushchekina S.V., Kharlamova A.D. (2015). 6-Methyluracil derivatives as bifunctional acetyl-cholinesterase inhibitors for the treatment of Alzheimer’s Disease. Chem. Med. Chem..

[14] Nurkenov O.A., Nurmaganbetov Z.S., Fazylov S.D., Seidakhmetova R.B., Shulgau Z.T., Muldakhmetov Z.M. (2022). Synthesis, structure and biological activity of (1*S*,9a*R*)-1*H*-1,2,3-triazol-1-yl)methyl)octahydro-1*H*-quinolizine derivatives of lupinine. Arch. Razi Inst..

[15] Marambio-Jones C., Hoek E.M.V. (2010). A review of the antibacterial affects of silver nanomaterials and potential implications for human health and the enironment. J. Nanopart. Res..

[16] Shameli K., Ahmad M.B., Zargar M., Yunus W.M.Z.W., Ibrahim N.A., Shabanzadeh P., Moghaddam M.G. (2011). Synthesis and characterization of silver/montmorillonite/chitosan bionanocomposites by chemical reduction method and their antibacterial activity. Int. J. Nanomed..

[17] Li W., Wang J., Chi H., Wei G., Zhang J., Dai L. (2012). Preparation and antibacterial activity of polyvinyl alcohol/regenerated silk fibroin composite fibers containing Ag nanoparticles. J. Appl. Polym. Sci..

[18] Caro C., Castillo P.M., Klippstein R., Pozo D., Zaderenko A.P. (2010). Silver nanoparticles: Sensing and imaging applications. Silver Nanoparticles.

[19] Prozorova G.F., Pozdnyakov A.S., Kuznetsova N.P., Korzhova S.A., Emel’yanov A.I., Ermakova T.G., Fadeeva T.V., Sosedova L.M. (2014). Green Synthesis of Water-Soluble Non-Toxic Polymeric Nanocomposites Containing Silver Nanoparticles. Int. J. Nanomed..

[20] Elliott C. (2010). The effects of silver sulfadiazine on chronic and burns wound healing. Br. J. Nurs..

[21] Rupp M.E., Fitzgerald T., Marion N., Helget V., Puumala S., Anderson J.R., Fey P.D. (2004). Effect of silver-coated urinary catheters: Efficacy, cost-effectiveness, and antimicrobial resistance. Am. J. Infect. Control.

[22] Cho K.H., Park J.E., Osaka T., Park S.G. (2005). The study of antimicrobial activity and preservative effects of nanosilver ingredient. Electrochim. Acta..

[23] Chou W.L., Yu D.G., Yang M.C. (2005). The preparation and characterization of silver-loading cellulose acetate hollow fiber membrane for water treatment. Polym. Adv. Technol..

[24] Sun R.W., Chen R., Chung N.P., Ho C.M., Lin C.L., Che C.M. (2005). Silver nanoparticles fabricated in Hepes buffer exhibit cytoprotective activities toward HIV-1 infected cells. Chem. Commun..

[25] Luo C.C., Zhang Y.H., Zeng X.W., Zeng Y.W., Wang Y.G. (2005). The role of poly(ethylene glycol) in the formation of silver nanoparticles. J. Colloid Interface Sci..

[26] Valencia G.A., Vercik L.C.O., Cilla T., Vercik A. (2012). A Simple and Green Method for Syhthesis of Ag and Au Nanoparticles using Biopolymers and Sugar as Reducing Agent. Mater. Res. Soc. Symp. Proc..

[27] Husen A., Siddiqi K.S. (2014). Phytosynthesis of nanoparticles: Concept, controversy and application. Nano Res. Lett..

[28] Bahavarnia F., Hasanzadeh M., Bahavarnia P., Shadjou N. (2024). Advancements in application of chitosan and cyclodextrins in biomedicine and pharmaceutics: Resent progress and future trends. RVS Adv..

[29] Ke Y., Junfeng L., Laichun L., Meilin L., Tanfang X., Junfen Z. (2023). Synthesis of cationic b-cyclodextrin functionalized silver nanoparticles and their drug-loading applications. RSC Adv..

[30] Fazylov S.D., Nurkenov O.A., Nurmaganbetov Z.S., Sarsenbekova A.Z., Bakirova R.Y., Seilkhanov O.T., Sviderskiy A.K., Syzdykov A.K., Mendibayeva A.Z. (2025). Synthesis of β-Cyclodextrin-Functionalized Silver Nanoparticles and Their Application for Loading Cytisine and Its Phosphorus Derivative. Molecules.

[31] Šesták J., Kratochvíl J. (1973). Rational approach to thermodynamic rrocesses and constitutive equations in isothermal and non-isothermal kinetics. J. Therm. Anal..

[32] Serra R., Nomen R., Sempere J. (1998). The non-parametric kinetics a new method for the kinetic study of thermoanalytical data. J. Therm. Anal. Calorim..

[33] Friedman H.L. (1964). Kinetics of Thermal Degradation of Char-Forming Plastics from Thermogravimetry. Application to a Phenolic Plastic. J. Polym. Sci. Part C Polym. Symp..

[34] Ozawa T.A. (1965). New Method of Analyzing Thermogravimetric Data. Bull. Chem. Soc. Jpn..

[35] Wenzler E., Maximos M., Asempa T.E., Biehle L., Schuetz A.N., Hirsch E.B. (2023). Antimicrobial susceptibility testing: An updated primer for clinicians in the era of antimicrobial resistance: Insights from the Society of Infectious Diseases Pharmacists. Pharmacotherapy.

[36] Barrot M. (2012). Tests and models of nociception and pain in rodents. Neuroscience.

[37] Meyer B.N., Ferrigni N.R., Putnam J.E., Jacobsen L.B., Nichols D.E., McLaughlin J.L. (1982). Brine Shrimp: A Convenient General Bioassay for Active Plant Constituents. Planta Med..

[38] McLaughlin J.L., Hostettmann K. (1991). Crown-gall tumours in potato discs and brine shrimp lethality: Two simple bioassays for higher plant screening and fractionation. Methods in Plant Biochemistry.

[39] Council N.R. (2011). Guide for the Care and Use of Laboratory Animals.

[40] Adekenov S.M., Zhanimkhanova P.Z., Nurmaganbetov Z.S., Amanzhan A., Chernov S.V., Turmukhambetov A.Z., Bagryanskaya I.Y., Gatilov Y.V., Shults E.E. (2019). Synthetic modifications of carboline alkaloid harmine: Synthesis of 8-substituted derivatives. Chem. Heterocycl. Compd..

[41] Nurmaganbetov Z.S., Fazylov S.D., Nurkenov O.A., Sarsenbekova A.Z., Pustolaikina I.A., Seilkhanov O.T., Sviderskiy A.K., Minayeva Y.V. (2025). Combined Computational and Experimental Study of Inclusion Complexes of Lupinine and its 1,2,3-triazole Derivative with β-cyclodextrin. Eurasian Chem.-Technol. J..

