# Supplementary material for: Synthesis and Properties of Silver Nanoparticles Functionalized with β-Cyclodextrin and Their Loading with Lupinine and Its Acetyl Derivatives
Source: Molecules. 2025 Aug 12;30(16):3354. doi: 10.3390/molecules30163354 (PMC12388491; doi:10.3390/molecules30163354)
Supplement: Supplementary file 1 [file molecules-30-03354-s001.zip › molecules-3752207-supplementary.pdf]

Synthesis and Properties of Silver Nanoparticles Functionalized with β-Cyclodextrin and Their Loading with Lupinine and Its Acetyl Derivatives

Serik D. Fazylov [1], Zhangeldy S. Nurmaganbetov [1,*], Oralgazy A. Nurkenov [1], Akmaral Z. Sarsenbekova [2], Olzhas T. Seilkhanov [3], Roza B. Seidakhmetova [4], Anel Z. Mendibayeva [1], Ryszhan Y. Bakirova [5] and Zainulla M. Muldakhmetov [1]

1. Laboratory of Synthesis of Biologically Active Substances, Institute of Organic Synthesis and Coal Chemistry of the Republic of Kazakhstan, Karaganda 100008, Kazakhstan; iosu8990@mail.ru (S.D.F.); nurkenov_oral@mail.ru (O.A.N.); anenyawa@mail.ru (A.Z.M.); iosu.rk@mail.ru (Z.M.M.)
2. Department of Physical and Analytical Chemistry, Karaganda University of the Name of E.A. Buketov, Karaganda 100074, Kazakhstan; chem_akmaral@mail.ru
3. Laboratory of Engineering Profile NMR-Spectroscopy, Sh. Ualikhanov Kokshetau University, Kokshetau 120000, Kazakhstan; seilkhanov@mail.ru
4. Department of Clinical Pharmacology and Evidence-Based Medicine, Karaganda Medical University, Karaganda 100012, Kazakhstan; rozabat@mail.ru
5. Department of Internal Diseases, Karaganda Medical University, Karaganda 100012, Kazakhstan; bakir15@mail.ru

* Correspondence: nzhangeldy@yandex.ru

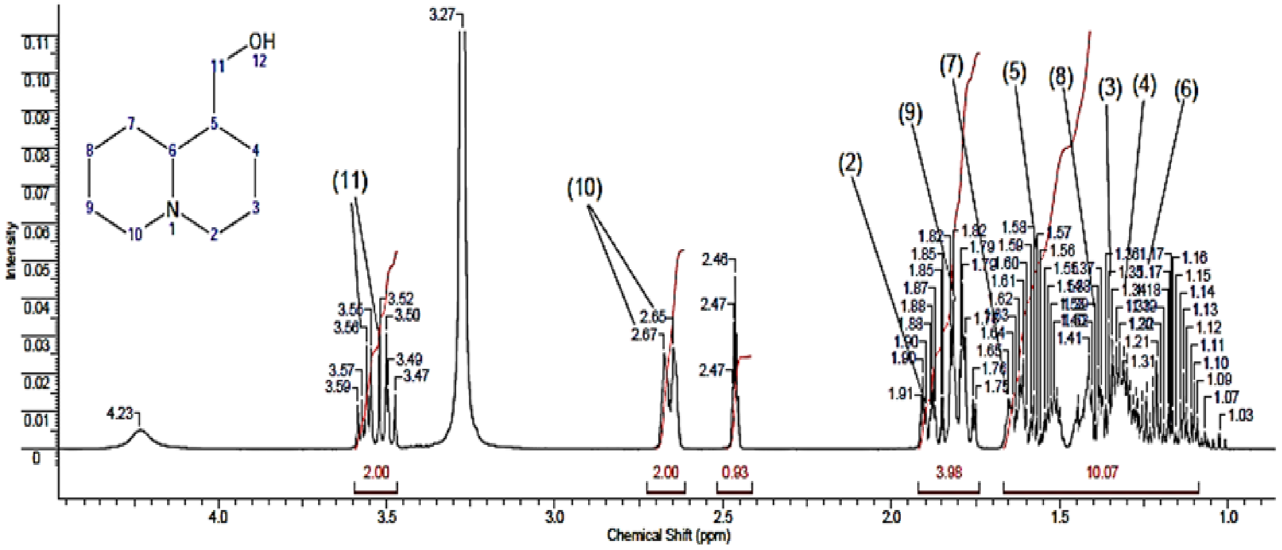

(a)

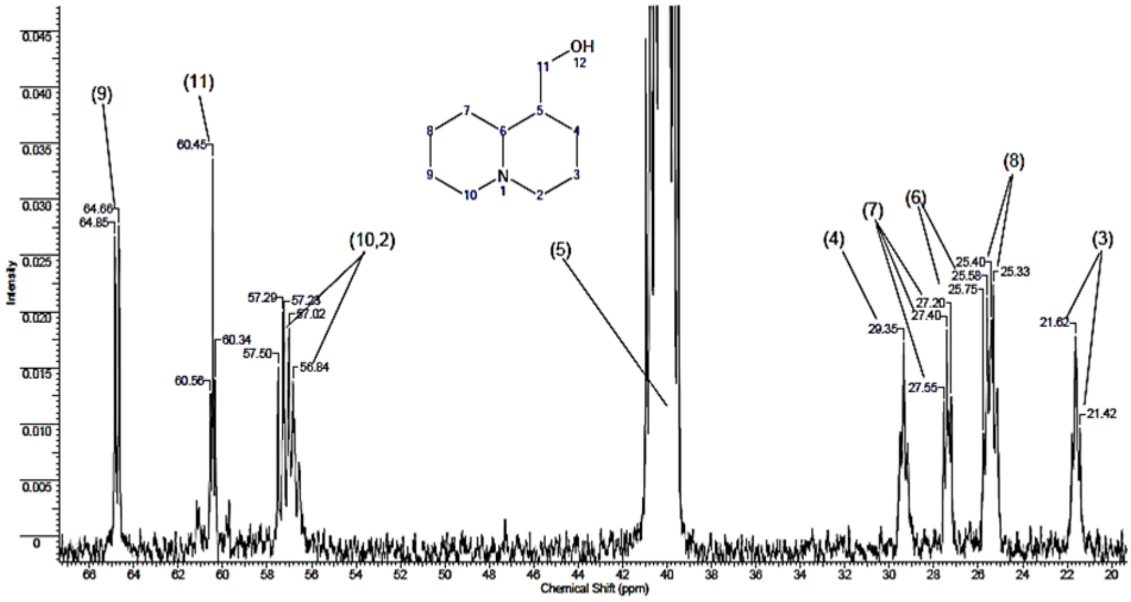

OH
12
11
7
5
8
6
4
9
N
3
10
1
2
(9)
(11)
(10,2)
(5)
(4)
(7)
(6)
(8)
(3)
64.66
64.85
60.45
60.34
60.56
57.29
57.23
57.02
57.50
56.84
29.35
27.55
27.20
27.40
25.40
25.58
25.75
25.33
21.62
21.42
Intensity
Chemical Shift (ppm)

(b)

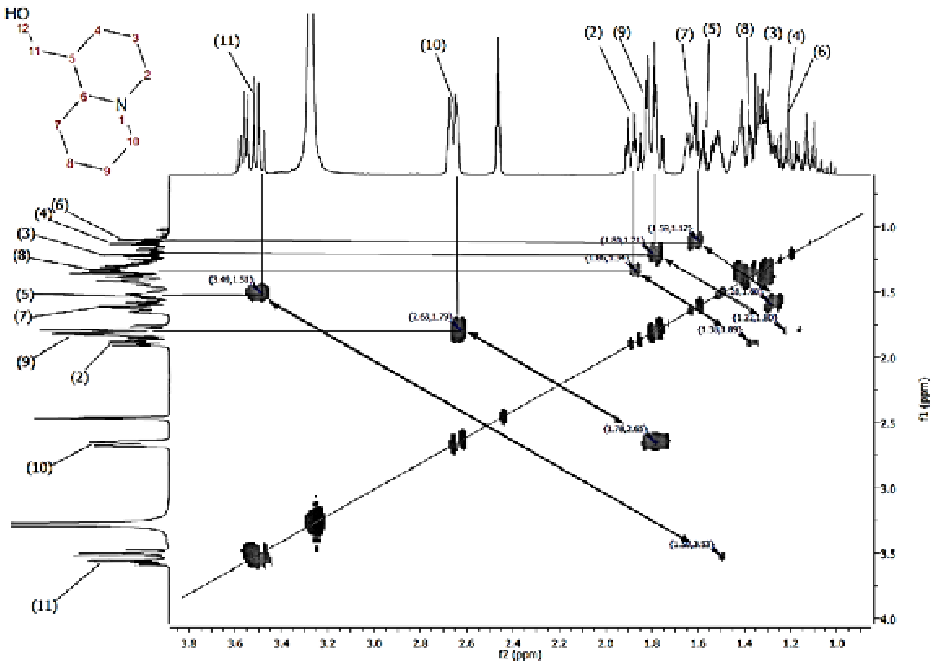

HO
12
11
(11)
(10)
(2)
(9)
(7)
(5)
(8)
(3)
(4)
(6)
f2 (ppm)
f1 (ppm)

(c)

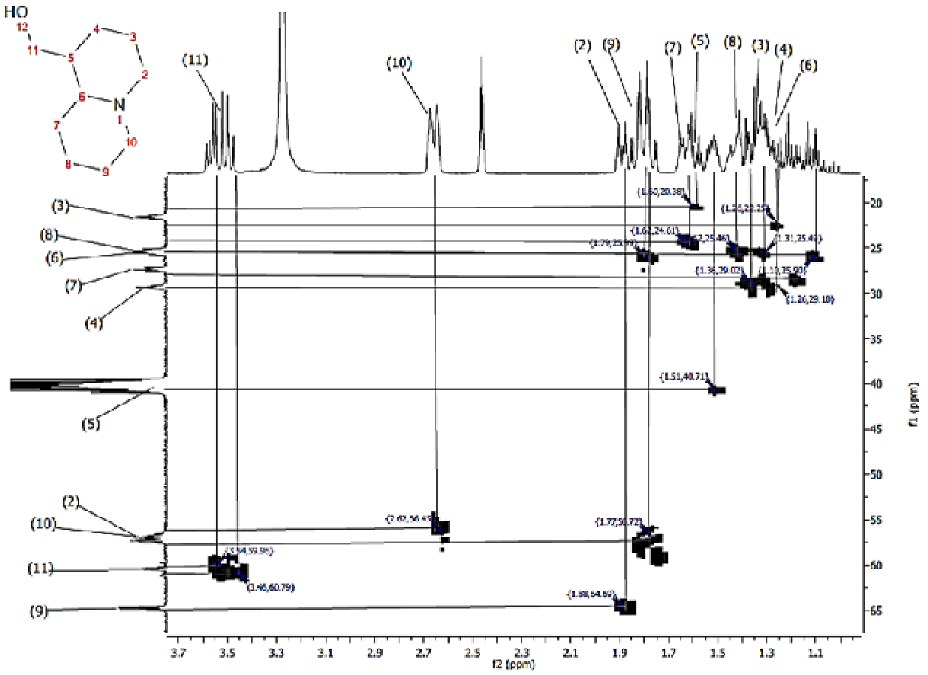

(d)

Figure S1. ^{1}H (a), ^{13}C (b), COSY (^{1}H-^{1}H) (c) and HMQC (^{1}H-^{13}C) (d) NMR spectras of lup

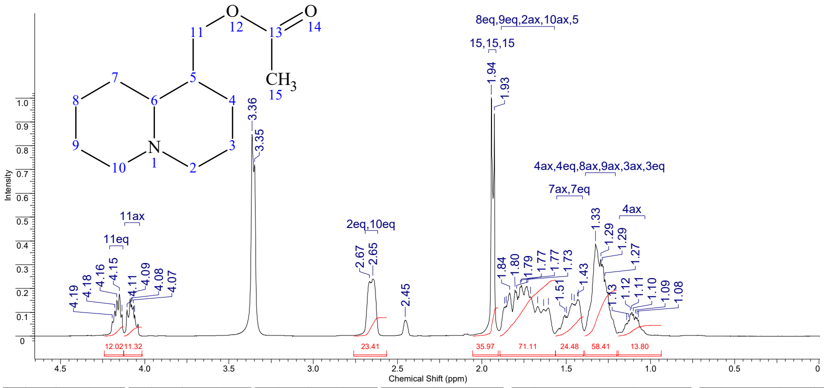

No.	(ppm)	(Hz)	Height
1	1.07	428.2	0.0649
2	1.08	431.4	0.0769
3	1.09	435.0	0.0811
4	1.10	440.5	0.0914
5	1.11	444.2	0.0998
6	1.12	447.4	0.0937
7	1.13	451.5	0.0653
8	1.14	456.5	0.0541
9	1.27	507.8	0.2374
10	1.29	514.2	0.3022
11	1.29	517.5	0.3212
12	1.33	529.8	0.3860

No.	(ppm)	(Hz)	Height
13	1.43	571.0	0.1525
14	1.43	572.9	0.1529
15	1.45	580.6	0.1344
16	1.47	586.6	0.1373
17	1.50	599.0	0.0822
18	1.51	602.6	0.0878
19	1.61	642.0	0.1181
20	1.63	653.5	0.1122
21	1.67	667.2	0.1248
22	1.71	684.1	0.1652
23	1.73	692.8	0.2098
24	1.77	705.7	0.2121

No.	(ppm)	(Hz)	Height
25	1.77	708.0	0.2070
26	1.79	717.6	0.1848
27	1.80	720.8	0.1925
28	1.84	733.6	0.1813
29	1.86	742.3	0.1267
30	1.87	746.0	0.1227
31	1.93	769.8	0.9350
32	1.94	777.1	1.0000
33	2.45	981.4	0.0668
34	2.65	1057.8	0.2404
35	2.67	1066.1	0.2296
36	3.35	1338.6	0.7176

No.	(ppm)	(Hz)	Height
37	3.36	1344.5	0.8484
38	4.06	1622.9	0.0880
39	4.07	1626.1	0.1242
40	4.08	1630.7	0.1313
41	4.09	1633.9	0.1543
42	4.11	1641.3	0.1067
43	4.14	1653.2	0.0901
44	4.15	1659.1	0.1762
45	4.16	1665.1	0.1485
46	4.18	1670.1	0.1048
47	4.19	1676.1	0.0606

No.	Annotation	(ppm)
1	4ax	[1.03 .. 1.18]
2	4ax,4eq,8ax,9ax,3ax,3eq	[1.21 .. 1.39]
3	7ax,7eq	[1.40 .. 1.56]
4	8eq,9eq,2ax,10ax,5	[1.57 .. 1.89]
5	15,15,15	[1.92 .. 1.96]
6	2eq,10eq	[2.62 .. 2.69]
7	11ax	[4.03 .. 4.12]
8	11eq	[4.13 .. 4.21]

No.	(ppm)	Value	Absolute Value
1	[4.02 .. 4.12]	11.322	1.11909e+1
2	[4.13 .. 4.24]	12.021	1.18818e+1
3	[0.94 .. 1.19]	13.795	1.36354e+1
4	[2.56 .. 2.76]	23.409	2.31381e+1
5	[1.40 .. 1.56]	24.479	2.41959e+1
6	[1.90 .. 2.05]	35.966	3.55493e+1
7	[1.20 .. 1.39]	58.408	5.77317e+1
8	[1.56 .. 1.89]	71.115	7.02912e+1

(a)

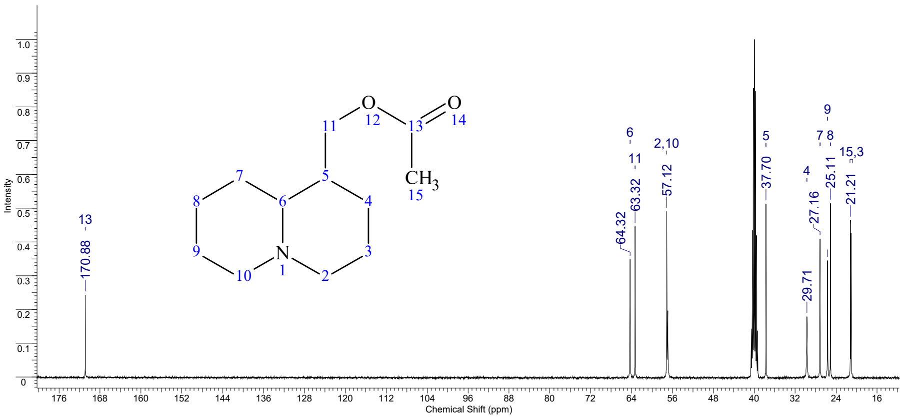

No.	(ppm)	(Hz)	Height
1	21.21	2131.8	0.4643
2	25.11	2523.8	0.5141
3	25.68	2581.3	0.3444
4	27.16	2729.8	0.4089
5	29.71	2986.7	0.1789
6	37.70	3789.9	0.5124
7	57.12	5742.3	0.4900
8	63.32	6365.3	0.4461
9	64.32	6465.9	0.3480
10	170.88	17177.8	0.2426

No.	Annotation	(ppm)
1	15,3	[20.77 .. 21.28]
2	8	25.11
3	9	25.68
4	7	27.16
5	4	29.71
6	5	37.70
7	2,10	57.12
8	11	63.32
9	6	64.32
10	13	170.88

(b)

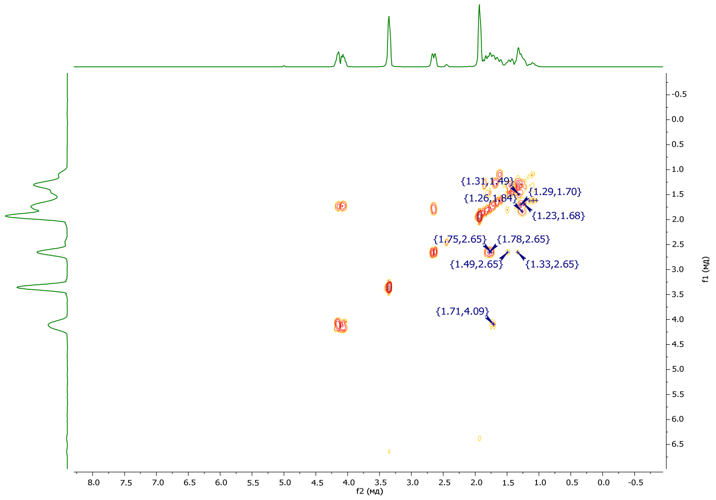

(c)

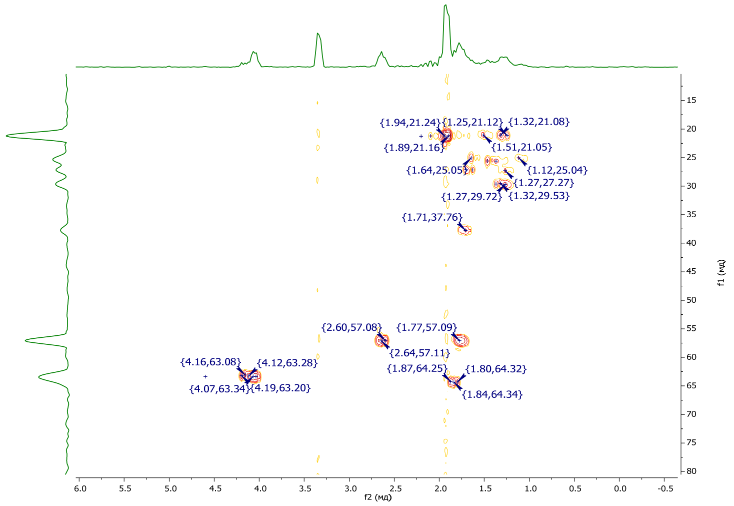

(d)

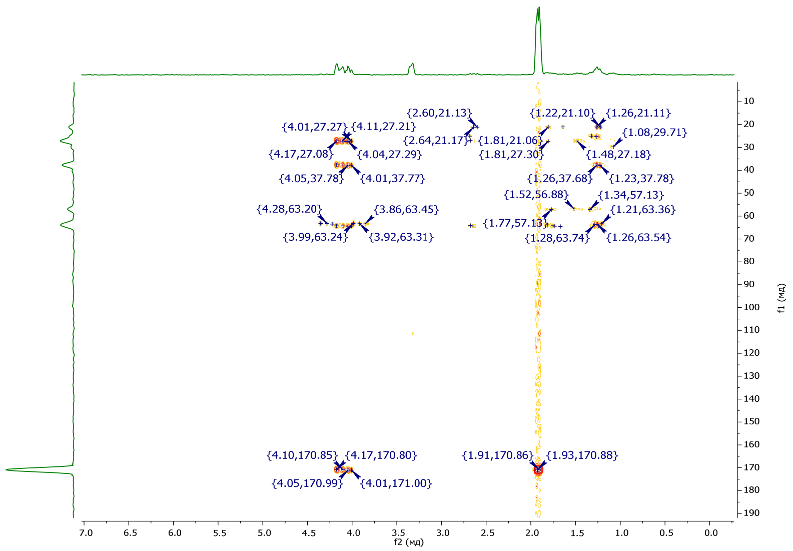

(e)

Figure S2. ^{1}H (a), ^{13}C (b), COSY (^{1}H-^{1}H) (c), HMQC (^{1}H-^{13}C) (d) and HMBC NMR spectras of lupacetat

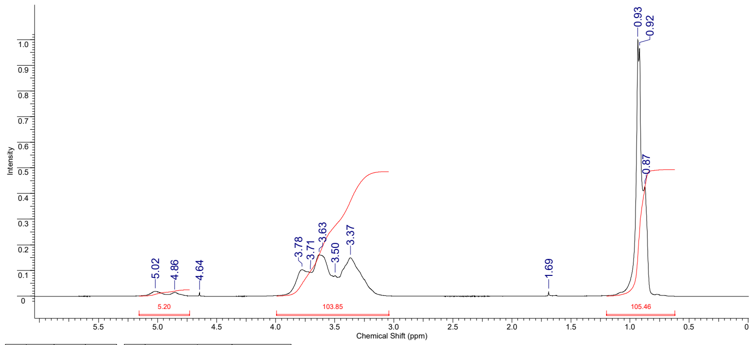

No.	(ppm)	(Hz)	Height
1	0.87	349.4	0.4267
2	0.92	367.2	0.9657
3	0.93	373.2	1.0000
4	1.69	675.0	0.0174
5	3.37	1346.3	0.1493
6	3.50	1397.6	0.0795
7	3.63	1452.1	0.1608
8	3.71	1481.4	0.0925
9	3.78	1510.3	0.1029
10	4.64	1856.9	0.0130
11	4.86	1941.7	0.0139
12	5.02	2006.7	0.0183

No.	(ppm)	Value	Absolute Value
1	[4.73 .. 5.16]	5.200	5.47764e+0
2	[3.04 .. 3.99]	103.846	1.09391e+2
3	[0.62 .. 1.20]	105.463	1.11094e+2

(a)

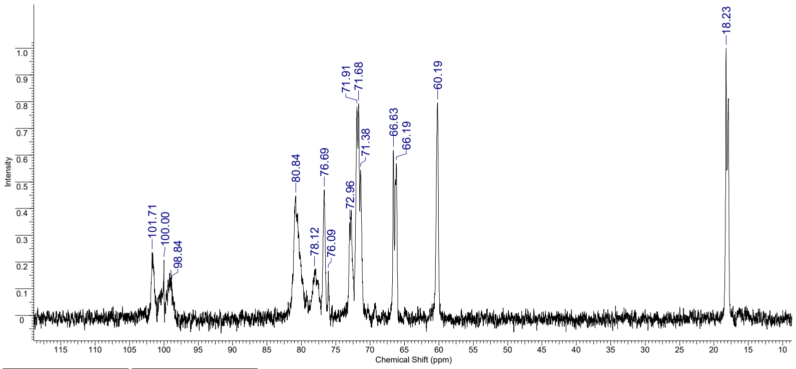

No.	(ppm)	(Hz)	Height	No.	(ppm)	(Hz)	Height
1	18.23	1832.7	1.0000	9	76.09	7648.7	0.1657
2	60.19	6050.9	0.7959	10	76.69	7709.1	0.4712
3	66.19	6653.8	0.5692	11	78.12	7852.8	0.1723
4	66.63	6697.9	0.6187	12	80.84	8126.0	0.4477
5	71.38	7175.2	0.5437	13	98.84	9935.6	0.1255
6	71.68	7205.9	0.7920	14	100.00	10052.5	0.2080
7	71.91	7228.9	0.7810	15	101.71	10224.1	0.2363
8	72.96	7334.3	0.3479				

b)

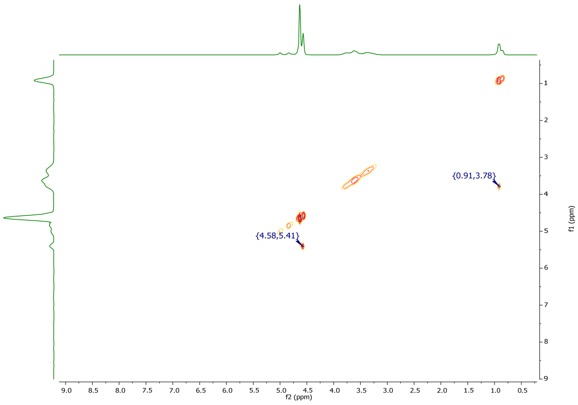

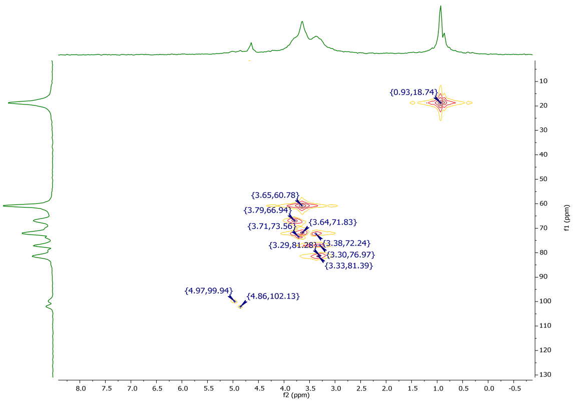

(c) (d)

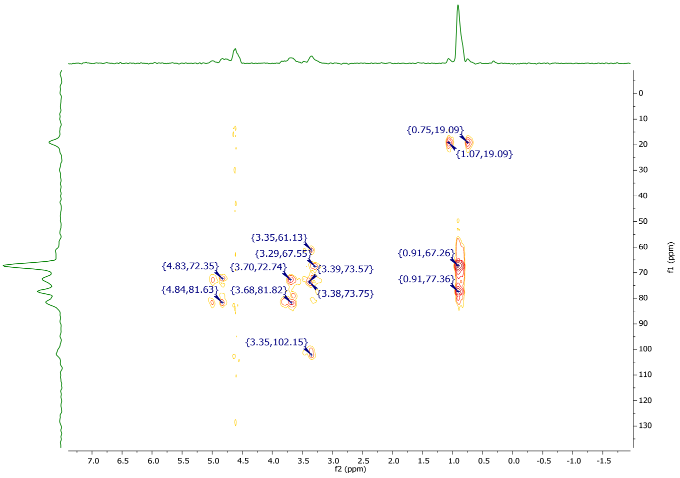

(e)

Figure S3. ^{1}H (a), ^{13}C (b), COSY (c), HMQC (d), HMBC (e) NMR spectra of 2gβCD-AgNPs (D_2O)

(a)

Stage 1
Initiation - beginning of degradation

Lup ⟶ R• + R′•

↓

Stage 2
decomposition of the oxazinine ring (T≈220-230°C)

$R\text{-}CH_2\text{-}CH\bullet \longrightarrow R\bullet + CH_2 = CH_2$

↓

Stage 3
dehydrogenation and formation of aromatic fragments:

successive dehydrogenation reactions ⟶ formation of cyclic and aromatic

↓

Stage 4

coking (condensation of residues):

condensation of carbonaceous residues leading to the formation of **pyrocarbon (coke)**

by-products ammonia, HCN, CO, CO_2, NH_3

(b)

Stage 1

destruction of the acetyl group (T≈180-220°C):

Lac $\longrightarrow$ Lup + CH_3COOH

↓

Stage 2

decomposition of the oxazinine ring (T≈220-230°C)

Lup $\longrightarrow$ Imine + cyclic ketone + H_2O

↓

Stage 3

chain fragmentation and dehydration(T≈300-400°C)

Imine $\longrightarrow$ NH_3 + aldehyde/ketone + CO_2

↓

Stage 4

pyrolysis of residual products (T>400°C)

residual fragments $\longrightarrow$ coke + gaseous products (H_2, CH_4)

(c)

Stage 1

Dehydration (to~120-150°C)

Lup-2gβ-CD-AgNPs ·H_2O $\longrightarrow$ Lup-2gβ-CD-AgNPs ·$H_2O\uparrow$

↓

Stage 2

decomposition of β-cyclodextrin (~200-300°C)

β -CD $\longrightarrow$ futural + CO_2 + low molecular weight

↓

Stage 3

destruction of lupinine (~250-350°C)

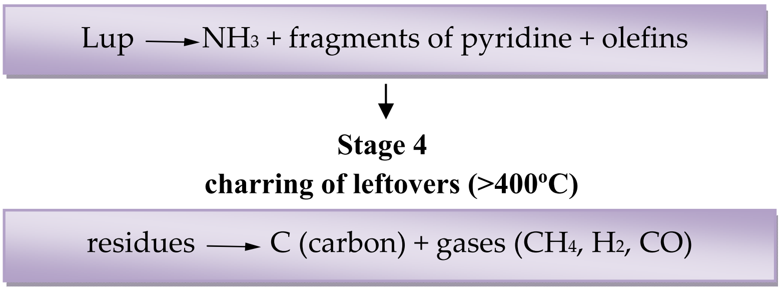

Figure S4. The proposed mechanisms of thermal decomposition are (a)—Lup; (b)—acetylated Lup; (c)—Lup inclusion complex with β-CD and silver nanoparticles (Lup-2gβCD-AgNPs). Stepwise breaking of molecular bonds leads to the formation of light volatile compounds and intermediate residues. In option (c), the process is altered due to the catalytic activity of silver nanoparticles

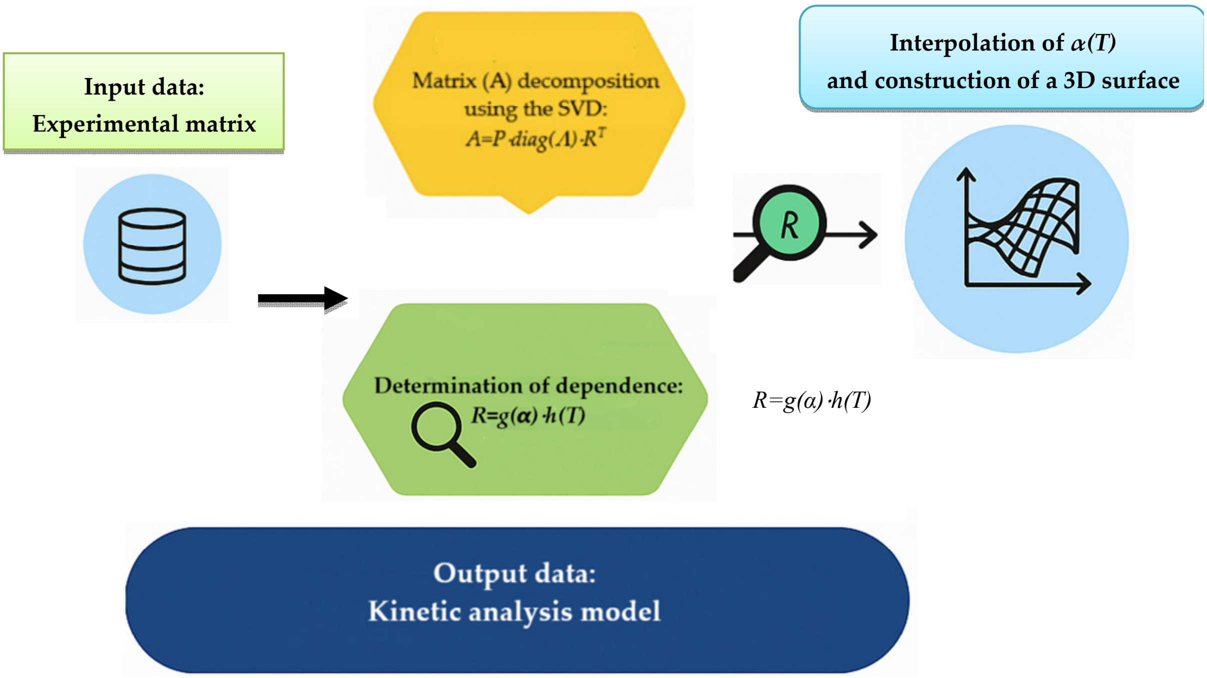

Where

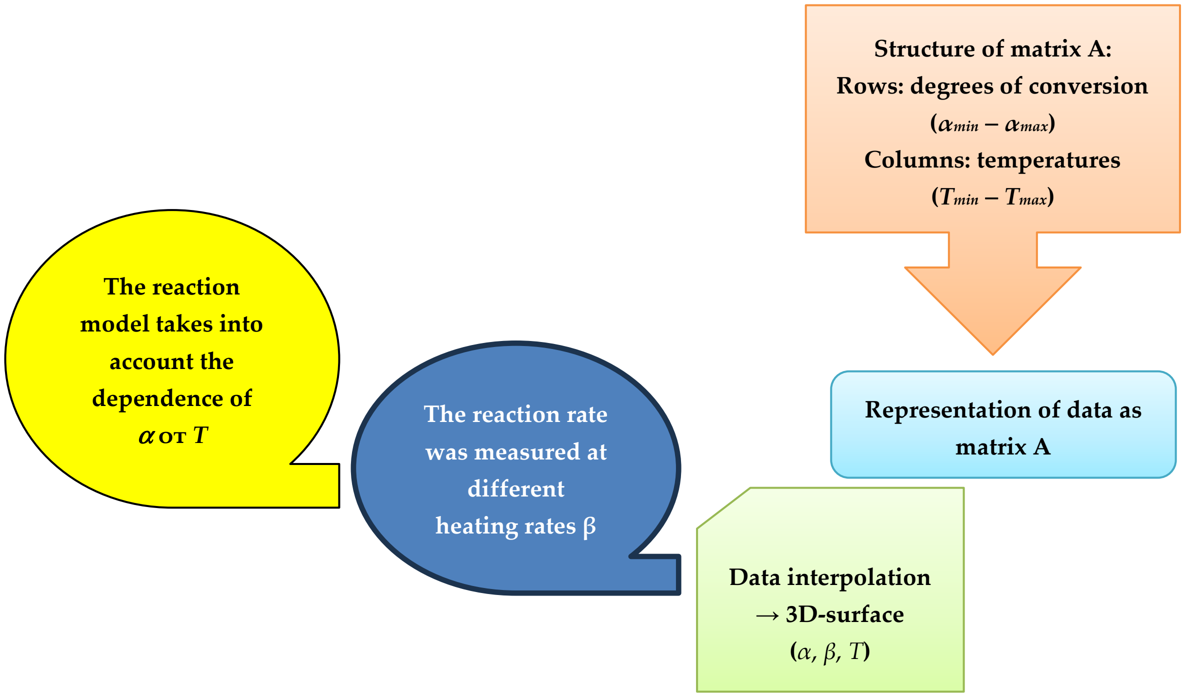

Figure S5. A block diagram of the stages of kinetic analysis using the method of model-free kinetics (NPK). The diagram shows the main stages of data processing: matrix formation, singular value decomposition (SVD), determination of the dependence $R = g(\alpha) \cdot h(T)$, interpolation and construction of a 3D surface, followed by the derivation of a kinetic model

Table S1. Antimicrobial activity of the samples

The cipher of the sample	*Staphylococcus aureus ATCC 6538*	*Bacillus subtilis ATCC 6633*	*Escherichia coli ATCC 25922*	*Pseudomonas aeruginosa ATCC 27853*	*Candida albicans ATCC 10231*
Lup-2gβCD-AgNPs	25	50	-	50	-
Lup-2gβCD	12.5	6.3	50	-	25
Lac	12.5	25	-	-	50
Lup	25	50	50	-	-
Control (ceftriaxone)	6.3	12.5	6.3	6.3	-
(Inappropriate)	-	-	-	-	12.5

The "–" sign indicates the absence of growth inhibition in the studied concentration range (1.56–50 micrograms/ml).

Table S2. Cytotoxic activity of the samples

Name of the substance	Concentration mcg/ml	Number of surviving larvae			LD_{50}, mcg/ml	Activity
		1st parallel	2nd parallel	3rd parallel		
Lup	1	9	8	8	74.6	+
	10	6	5	5		
	100	5	4	5		
Lup-2gβCD-AgNPs	1	7	7	7	54.3	+
	10	5	4	4		
	100	3	3	3		
Lac	1	8	8	8	68.5	+
	10	6	7	6		
	100	5	4	4		
Lup-2gβCD	1	8	7	8	62.6	+
	10	7	6	6		
	100	5	5	4		
Comparison drug: dactinomycin *(actinomycin D)*	1	6	7	7	47.8	-
	10	4	3	4		
	100	2	2	3		

Table S3. Analgesic activity of the samples

Name of the substance	Dose, mg/kg	Number of convulsions	Reduction of the number of vinegar crunches (%)
Control	-	102.6± 11.2	100
Diclofenac sodium	8	48.2± 9.6	53.0
Lup-2gβCD-AgNPs	25	59.1± 10.4	42.4
Lup-2gβCD	25	72.4±10.8	29.4
Lac	25	43.6 ±9.4*	57.5
Lup	25	83.5 ± 10.1*	18.6

Note: * – $p<0.05$ compared to the control
